# Guide posts for investment in primary health care and projected resource needs in 67 low-income and middle-income countries: a modelling study

**DOI:** 10.1016/S2214-109X(19)30416-4

**Published:** 2019-09-26

**Authors:** Karin Stenberg, Odd Hanssen, Melanie Bertram, Callum Brindley, Andreia Meshreky, Shannon Barkley, Tessa Tan-Torres Edejer

**Affiliations:** a Department of Health Systems Governance and Financing, WHO, Geneva, Switzerland; b Swiss Tropical and Public Health Institute, Basel, Switzerland; c University of Basel, Basel, Switzerland; d Independent consultant, Geneva, Switzerland; e Department of Integrated Health Services, WHO, Geneva, Switzerland

## Abstract

**Background:**

Primary health care (PHC) is a driving force for advancing towards universal health coverage (UHC). PHC-oriented health systems bring enormous benefits but require substantial financial investments. Here, we aim to present measures for PHC investments and project the associated resource needs.

**Methods:**

This modelling study analysed data from 67 low-income and middle-income countries (LMICs). Recognising the variation in PHC services among countries, we propose three measures for PHC, with different scope for included interventions and system strengthening. Measure 1 is centred on public health interventions and outpatient care; measure 2 adds general inpatient care; and measure 3 further adds cross-sectoral activities. Cost components included in each measure were based on the Declaration of Astana, informed by work delineating PHC within health accounts, and finalised through an expert and country validation meeting. We extracted the subset of PHC costs for each measure from WHO's Sustainable Development Goal (SDG) price tag for the 67 LMICs, and projected the associated health impact. Estimates of financial resource need, health workforce, and outpatient visits are presented as PHC investment guide posts for LMICs.

**Findings:**

An estimated additional US$200–328 billion per year is required for the various measures of PHC from 2020 to 2030. For measure 1, an additional $32 is needed per capita across the countries. Needs are greatest in low-income countries where PHC spending per capita needs to increase from $25 to $65. Overall health workforces would need to increase from 5·6 workers per 1000 population to 6·7 per 1000 population, delivering an average of 5·9 outpatient visits per capita per year. Increasing coverage of PHC interventions would avert an estimated 60·1 million deaths and increase average life expectancy by 3·7 years. By 2030, these incremental PHC costs would be about 3·3% of projected gross domestic product (GDP; median 1·7%, range 0·1–20·2). In a business-as-usual financing scenario, 25 of 67 countries will have funding gaps in 2030. If funding for PHC was increased by 1–2% of GDP across all countries, as few as 16 countries would see a funding gap by 2030.

**Interpretation:**

The resources required to strengthen PHC vary across countries, depending on demographic trends, disease burden, and health system capacity. The proposed PHC investment guide posts advance discussions around the budgetary implications of strengthening PHC, including relevant system investment needs and achievable health outcomes. Preliminary findings suggest that low-income and lower-middle-income countries would need to at least double current spending on PHC to strengthen their systems and universally provide essential PHC services. Investing in PHC will bring substantial health benefits and build human capital. At country level, PHC interventions need to be explicitly identified, and plans should be made for how to most appropriately reorient the health system towards PHC as a key lever towards achieving UHC and the health-related SDGs.

**Funding:**

The Bill & Melinda Gates Foundation.

## Introduction

Primary health care (PHC) is globally acknowledged as an essential driving force for advancing universal health coverage (UHC) and the 2030 Agenda for Sustainable Development.^1^ Investing in PHC yields high returns and promotes sustainability.^2^ Yet, only half of government spending in health across low-income and middle-income countries (LMICs) is currently allocated to primary care and public health interventions—ie, PHC services.^3^ Ensuring quality PHC services should not be considered a low-cost strategy: investing in accessible health systems that provide a comprehensive set of interventions to all those in need can bring enormous benefits but will require significant financial investments. Among the commitments made at the Global Conference on Primary Health Care in October, 2018, countries and other stakeholders pledged to ensure adequate financing of primary health care in the Declaration of Astana.^4^ The definition of how much is adequate is subject to debate.

**Research in context**

**Evidence before this study**

Although the role of primary health care (PHC) for health outcomes is widely acknowledged, substantial variation exists in health service models and packages among countries, and little work has been done to develop detailed definitions of what PHC encompasses. Similarly, as of yet, few global benchmarks have been proposed for health system investment to strengthen PHC. Several studies and initiatives have considered the resource implications of functioning PHC systems. For example, in 1986, Patel suggested that PHC costs across developing countries would amount to around 5% of gross domestic product, but also noted that PHC programmes are largely undefined in terms of their content. Although the policy debate and visibility of PHC has increased, the boundaries of PHC, as well as related budgetary needs, remain poorly conceptualised. The Primary Health Care Performance Initiative was established in 2015 to support measurement of PHC-related variables, with PHC expenditure per capita as one recommended indicator. Country scorecards were designed, with an intent to report current PHC expenditure using a standardised method but without identifying a target. At the global level, Watkins and colleagues estimated the total annual cost of providing essential PHC interventions across low-income countries and lower-middle-income countries at 80% population coverage to be about $350 billion, or about $97 per capita on average (in 2016 US$). Although global per capita estimates of service delivery are useful to provide a rough indication of spending needs, countries also need guidance on system requirements to expand PHC. Therefore, our study takes an ingredients-based approach to be able to identify the specific inputs needed to strengthen PHC across different country contexts.

**Added value of this study**

To the best of our knowledge we develop here, for the first time, three measures that relate to investments to strengthen PHC-orientation in health systems, identifying both specific interventions and health systems requirements. The added value of our study is that we discuss different measures of PHC costs; we specify the inputs required to strengthen PHC delivery across countries and estimate costs using country-specific data; we present and discuss related investment guide posts; and we add to the evidence base on the potential impact to be brought by investments in PHC as well as the affordability of delivering high-quality PHC across different settings.

**Implications of all the available evidence**

Defined service packages, emphasising primary care and public health services, as well as multisectoral actions with an impact on health, help inform the health investment agenda in low-income and middle-income countries. The results provide evidence on the likely cost drivers within countries seeking to expand their health service coverage by strengthening PHC, and an estimate of additional resource needs. Impact modelling indicates that significant health gains can be achieved by investing in primary care and public health and confirms the catalytic role that PHC can play in the universal health care and Sustainable Development Goals agendas. Although all three measures presented here are important for operationalising PHC, countries can assess which measure is most relevant for their specific dialogue around investments and monitoring purposes, and use this information to drive context-appropriate investments along a continuum of comprehensive PHC.

Questions around spending targets for PHC have been debated for decades. In 1986, Patel^5^ suggested that advancing PHC across so-called developing countries would cost about 5% of gross domestic product (GDP). Because of the realisation that resources are constrained, some stakeholders opted for a more selective definition of PHC, emphasising a smaller set of highly cost-effective interventions.^6^ The past 10 years have seen a revitalisation of support for a broader PHC agenda, in line with the original commitments in the Declaration of Alma-Ata, and its importance in achieving the health-related Sustainable Development Goals (SDGs). The Primary Health Care Performance Initiative was established in 2015 to support measurement of PHC-related variables, with PHC expenditure per capita as one recommended indicator.^7^ Estimates of current PHC expenditure range between US$15 and $60 per capita in LMICs, depending on the definition applied.^3^ At the country level, information on current spending can be compared against needs-based targets and projected costs for expanding service delivery, to inform resource allocation decisions.

Global resource needs for the SDGs serve multiple purposes, including advancing our understanding of implementation strategies or production functions, supporting resource mobilisation, and informing financing strategies.^8^ Global PHC costs were estimated in a 2018 study based on a defined set of services delivered through selected platforms that serve as first point of contact.^9^ Watkins and colleagues^9^ thus estimated that total annual costs of delivering PHC interventions in low-income countries and lower-middle-income countries, at 80% population coverage, would amount to $350 billion, or about $97 per capita (2016). However, their approach did not explicitly consider that different countries have different capacities for scale-up and did not provide details on the type of system investments needed.

To adequately plan and finance health systems, and in particular to strengthen their PHC orientation, governments need information on the specific health system investments required. Here, we add to the global evidence base by drawing on the framework for WHO's previously published price tag for the health SDG targets^10^ and specifically identifying investments required for PHC across the health system building blocks. By using an ingredients-based approach we can allow for differentiated coverage targets across different categories of countries and identify the cost drivers for accelerating PHC implementation. Explicitly defined measures for PHC investment needs can be used to advocate for increased budget allocations.

## Methods

### Study design

This modelling study was done using data from 67 LMICs (appendix p 2). Three measures of PHC costs were developed using a functional definition that focuses on the purpose of PHC interventions, rather than where, how, or by whom they are delivered or were developed. This approach is consistent with others in the literature, such as the methodology used by WHO to estimate current PHC expenditure, based on the system of health accounts, which uses a functional classification of services provided to monitor PHC expenditure.^3^ Another advantage of this approach is that the explicit definition of PHC interventions within a country's essential service package can be aligned with the principle of progressive realisation of UHC (ie, increasing access to an expanding PHC intervention package over time).^11^

Recognising the variation in PHC interventions among countries, we propose three measures of PHC, which represent a successive expansion of the scope of interventions (table 1). These three measures are consistent with global definitions of PHC as agreed at the Global Conference on Primary Health Care, which emphasised that the three components of PHC as primary care and essential public health functions are the core of integrated health services; multisectoral policies and actions; and empowered people and communities.^4^ Given the variation in health service models and intervention packages among countries, little work has been done to develop benchmarks for health system investment to strengthen PHC.^12^ Our proposed measures correlate with the definitions used within the system of health accounts to differentiate between specialised and non-specialised (general) care health services.^13^ The list of interventions included in each measure was further informed by an expert meeting and country validation process.

**Table 1:** Three proposed measures for PHC investment needs

	**Measure 1: focus on preventive and outpatient care as the basis for PHC**	**Measure 2: expanded measure considering general inpatient care and supportive health systems**	**Measure 3: broader PHC measure including cross-sectoral investment**
Description	This measure centres on preventive and outpatient care. Preventive interventions incorporate public health interventions such as behaviour change, policy, and tax interventions when aimed at adjusting behaviour. Outpatient care is limited to non-specialist outpatient care services, using definitions commonly applied within a health accounts framework. This measure also includes a share of the required resources for information systems, good governance and financing.	This measure adds general inpatient care, orthopaedic devices, and prosthetics; full health sector cost for strengthening information systems, good governance, and financing; and the cost of health emergency preparedness (and compliance with the International Health Regulations).	This measure captures broader cross-sectoral investments important for advancing PHC, including investments in water, sanitation, and hygiene; indoor air pollution; and food safety. This measure also considers essential information-gathering functions, done only in part by the health sector, such as censuses and civil registry systems.
Rationale	This measure is aligned with a common understanding that PHC primarily reflects first contact at lower (close-to-patient) levels of the health system and should focus on preventive care and general outpatient care; it thereby centres on the essential components of PHC in promoting preventive and close-to-client services	This measure captures a broader interpretation of the Alma-Ata declaration and considers skilled care at birth, which otherwise would not be covered under PHC; the boundaries for this measure remain limited to the health sector	This measure responds to the broad SDG agenda and the need to consider cross-sectoral investments as PHC is advanced in the 21st century; the boundaries are expanded beyond the health sector to include key cross-sectoral interventions
Consistency with expenditure monitoring	The measure is consistent with the health-care based approaches put forward by Van de Maele and colleagues^3^ for monitoring PHC expenditure	This measure goes beyond current proposed approaches for monitoring expenditure on PHC, which do not include inpatient care when delivered in hospitals	This measure goes beyond current proposed approaches for monitoring expenditure on PHC using health accounts, which by necessity are limited to the health sector only
Health intervention components	Population-based interventions; generalised outpatient care; medicines, diagnostic tests, and supplies; programme support costs	Population-based interventions; generalised outpatient care; generalised inpatient care; medicines, diagnostic tests, and supplies; orthopaedic devices and prosthetics; programme support costs	Population-based interventions; generalised outpatient care; generalised inpatient care; medicines, diagnostic tests, and supplies; orthopaedic devices and prosthetics; programme support costs; conditional cash transfers for demand generation; cross-sectoral interventions (eg, water, sanitation, and hygiene; road safety; violence reduction; pollution control; and food safety)
Functional service delivery systems components	Health workforce salaries and in-service training; health facility infrastructure construction, refurbishing, and maintenance; medical equipment purchase and maintenance; logistics and supply chain	Health workforce salaries and in-service training; health facility infrastructure construction, refurbishing, and maintenance; medical equipment purchase and maintenance; logistics and supply chain	Health workforce salaries and in-service training; health facility infrastructure construction, refurbishing, and maintenance; medical equipment purchase and maintenance; logistics and supply chain
Governance, financing, and monitoring components	Governance (share 80%); financing (share 80%); health information systems (some components); and laboratory capacity	Governance (full 100%); financing (full 100%); health information systems; health emergency preparedness; emergency response	Governance (full 100%); financing (full 100%); health information systems in the health sector and beyond; health emergency preparedness; emergency response

PHC=primary health care. SDG=Sustainable Development Goal.

Measure 1 includes preventive public health interventions and general outpatient services, and is thus largely consistent with the measure put forth for monitoring current expenditure as per the national health accounts framework.^3^ Measure 2 broadens the scope of interventions by including non-specialised inpatient services, including uncomplicated deliveries and immediate neonatal care; it also captures a broader range of costs related to supportive health systems and health security, including compliance with the international health regulations.^14^ Measure 3 goes further in capturing broader cross-sectoral investments that are important for health outcomes, such as water and sanitation, air quality improvements, road safety, and food safety, as well as broader health security (including animal health and zoonotic disease control). Specialised care, however, whether outpatient or inpatient, is excluded from all three measures. Cross-sectoral investments as included under measure 3—eg, investments to address respiratory, food-borne and water-borne diseases—play a key role that has been clearly established in the literature, and roughly a quarter of global disease burden could be prevented by reducing environmental risks.^15^ Importantly, our measures delink interventions from the level of care, insofar as we include costs and effects for PHC interventions, even when they might be delivered at higher levels of care.

### Applying PHC measures within a modelling framework

To apply these measures to an investment model, our starting point is WHO's cost projections for reaching the health SDG targets, published in 2017.^10^ An explicitly defined set of essential health interventions (a health benefits package) and associated health system investments necessary to achieve the health-related SDGs were modelled to be scaled up from 2016 to 2030, across 67 LMICs. Within the model, countries are assumed to invest over time according to the maturity of their system, thereby progressing at different speeds according to their current system strength and capacity. The WHO 2017 model was largely limited to the health sector (table 2).

**Table 2:** Applying the three PHC measures to WHO's SDG price tag model

		**Measure 1 (M1)**	**Measure 2 (M2)**	**Measure 3 (M3)**
**Health interventions**				
Number of health interventions considered as PHC (out of 188 interventions in original SDG model)		143	152	160
Examples of interventions included under each PHC measure, by platform				
	Policy and population-wide interventions	Legislative and regulatory interventions such as taxes on alcohol and tobacco, marketing restrictions, and bans; population-level behaviour change communication campaigns—eg, breastfeeding for infants and safe sex to reduce HIV transmission	Same as M1	Same as M1, plus water, sanitation, and hygiene interventions
	Periodic outreach and schedulable services	Vaccination programmes; family planning; nutrition counselling and micronutrient supplementation	Same as M1	Same as M1
	First-level clinical services	Disease-specific pharmaceutical treatment through outpatient care (eg, oral antibiotics for pneumonia, first-line tuberculosis treatment, standard glycaemic control treatment for diabetes); counselling and support for behaviour change (eg, smoking cessation)	Same as M1, plus normal delivery and basic neonate resuscitation	Same as M2
	Care provided at first level and above	Mammography to detect breast cancer; treatment of asthma and chronic obstructive pulmonary disease	Same as M1, plus basic emergency obstetric care	Same as M2
**Health system strengthening**				
Health workforce		Health workforce estimates are calculated for three categories: medical doctors, nurses or midwifes, and other. We use a bottom-up approach to estimate the full-time equivalent workers required to provide the defined package of PHC interventions, by country and by year. Bottom-up estimates were also calculated for the full SDG set of interventions and a relative share was subsequently estimated for PHC. The relative share was applied to the total number of health workers estimated to be required for the WHO SDG price tag, which was based on target population-density ratios. Using this approach, the estimated health worker cost for PHC is a proportion of the population-density-based cost as estimated in the SDG price tag.	Similar to M1, we calculate the share of health workers' time spent delivering PHC interventions within the context of the overall SDG price tag; under M2, the share is greater, because it includes more interventions than M1. In order to account for generalised inpatient care, we include an additional share of health worker time.	Same as M2
Infrastructure and equipment		The model includes costs for health centres, district hospitals, and provincial hospitals. The full costs of building, refurbishing, and maintaining health centres is attributed to PHC. For district hospitals and provincial hospitals, we include a percentage share of the cost required to construct, refurbish, and equip. The share is derived from national health accounts expenditure data on non-specialised outpatient care in low-income and middle-income countries (33% for district hospitals and 3% for provincial hospitals).	Similar to M1, we include the full cost of health centres. We increase the share of costs allocated to PHC from district hospitals to 81% to account for general (non-specialised) inpatient care, and similarly increase the share to 27% for provincial hospitals. Again, the shares are based on data from national health accounts.	Same as M2
Health information system		Costs for strengthening the health-facility-based system	Costs include components related to strengthening the health-facility-based system, administrative information systems, public health institutes, and administration of surveys	Same as M2, plus the full cost for a census and civil registry system (includes costs beyond the health sector)
Medicines, diagnostics, and supplies		Costs are directly estimated based on medicines, diagnostics, and supplies required for each intervention, multiplied by the numbers reached by country and year	Same as M1, plus a greater cost because more interventions are included	Same as M2, plus considering a greater number of interventions
Supply chain		The cost of supply chain was estimated by taking a share of the supply chain cost from the 2017 WHO SDG price tag; the share is based on the relative total cost of commodities provided under each PHC package, compared with the total cost of commodities estimated in the SDG price tag; costs for cold chain are estimated separately and included fully	Same as M1, plus considering the specific commodity costs for M2; cold chain is separate and included fully	Same as M2, plus considering the specific commodity costs for M3; cold chain is separate and included fully
Health financing		80% of health-financing-related costs are included	The full health-financing costs are included	Same as M2
Governance		80% of governance-related costs are included	The full governance costs are included	Same as M2
Emergency risk management or International Health Regulations		A share of laboratory costs at the district and provincial hospital level; the share applied is the same as for the infrastructure component	Same as M1, plus all costs for preparedness except poison control centres and national laboratories	Same as M2
Emergency relief (health worker hazard pay for working in distressed settings)		Included in their entirety	Included in their entirety	Included in their entirety
Facility reconstruction in post-conflict settings		Included in their entirety	Included in their entirety	Included in their entirety
Programme support costs		Included in their entirety for health sector	Included in their entirety for health sector	Included in their entirety for health sector plus additional costs for multisectoral HIV and AIDS interventions
Cash transfers to increase care seeking		Excluded	Included specifically for skilled birth attendance	Same as M2 plus included for general health-care seeking

M1=measure 1. M2=measure 2. M3=measure 3. PHC=primary health care. SDG=Sustainable Development Goal.

The WHO SDG projection model was designed with a PHC-centred approach in mind—ie, the health workforce and infrastructure models were set up to accommodate a close-to-client health system, which makes it appropriate for our analysis. However, the model focuses on health sector investments, which limits the completeness of resource needs for measure 3, which go beyond the health sector. The original SDG model included 188 specific health interventions. These were established either to be PHC interventions or not, according to their classification within the health accounts framework. All interventions delivered through population or outreach platforms were included in one or more PHC measures, as were most clinical services (appendix pp 7–18). Shared investments required across the health system were estimated for the different health system building blocks as defined by WHO. Here, we used a mix of methods, in which some allocation rules were adopted from the expenditure monitoring framework (eg, governance-related costs) and others were derived from the PHC-relevant share of overall service delivery estimated within the WHO SDG projection model (eg, the share of health workforce and supply chain costs for PHC services as a share of the full modelled package of interventions). To the extent possible, health system investments are directly related to the package of interventions considered under each measure. Costs are estimated by country and by year using an ingredients-based approach—ie, breaking each investment down into a multiplication of quantities (by year) by prices, and using country-specific prices when available (appendix p 5).

The baseline year in our model is 2015, with investments scaled up successively until 2030. Our estimates are thus incremental to the assumed 2015 investment levels, and we assume limited progress was made during 2015–19.^16^ Capital investment costs originally modelled for 2016–19 were redistributed to years 2020–30.

Here, we present incremental outputs for 2020–30. Similar to the previously published SDG projections, we present estimates for eight groups of countries: three income categories and five additional country categories representing different degrees of health system maturity (conflict, vulnerable, and health system strength 1, 2, 3; appendix p 4).

### Projecting impact

For most interventions, estimates of health impact are projected using the impact models within the OneHealth Tool. The models include number of deaths prevented, stillbirths prevented, HIV infections, and prevention and treatment of non-communicable diseases and mental health disorders resulting from expanding coverage of PHC interventions. For a few areas (tuberculosis, neglected tropical diseases [NTDs], and cervical cancer screening), impacts were estimated outside of the OneHealth Tool and added in manually. To project gains in life expectancy we developed standard life tables using the standard UN life table methods for the base year of 2015 and the final year of 2030.^17^ For the life table input of number of deaths in 2030, we subtracted deaths avoided for tuberculosis, NTDs, and cervical cancer screening from the deaths calculated in the OneHealth Tool to estimate the total number of deaths by age and sex for each country included in the analysis. We then estimated life expectancy at birth for each country in 2030 and calculated the difference between 2030 and 2015. Healthy life-years gained are calculated by comparing the healthy years of life lived between the population in flatline projections (ie, unchanged coverage) and the PHC scale-up scenarios. Health adjustments are made using the Global Burden of Disease disability weights for each health state modelled. In addition to those conditions modelled in the OneHealth Tool, we add healthy life-years for tuberculosis,^18^ NTDs,^19^ and stillbirths avoided.^10^

### Validation process

The approach to define PHC measures was discussed with country representatives and global health policy and modelling experts in a meeting in Geneva on Sept 24–27, 2018. Participants included international experts and academics, as well as representatives from 12 LMICs whose population jointly accounts for more than 48% of the population in the 67 countries covered in the model. Country-specific data inputs used for the modelling were reviewed and validated by country participants during the meeting, as well as through follow-up correspondence. Data were provided by country participants for the most recent year available.

### Developing PHC investment guide posts

Investment guide posts are derived from our model for costs, outpatient visits, and health workforce. The estimates are generated by country-specific models but are aggregated and presented as average estimates by income level and country type. Guide posts are a partial measure, covering only the modelled interventions. Here, we present them as conceptual measures for future assessment and monitoring purposes.

Projected costs for PHC are presented as additional and total per capita estimates (panel). The additional amounts represent what is required to advance health service delivery beyond current levels of investment, assuming that current levels of investment remain at a constant level.

Panel **Constructing investment guide posts for total primary health care (PHC) expenditure**

Total PHC cost per capita is calculated as the sum of estimated current PHC expenditure plus the additional amount estimated through our analysis. Current PHC expenditure is estimated by applying option 5 from van de Maele and colleagues,^3^ which includes general outpatient care, medical goods, and a share of administration costs. Data on current spending was available from WHO's global health expenditure database in 2016 US$ for 45 low-income and middle-income countries. We deflated estimates to 2014 US$ using country-specific price deflators. Results are presented as population-weighted average values by country group.

Expenditure estimates on PHC from health accounts only include recurrent expenditures, since expenditure on capital goods is reported separately within health accounts and no proposed methods exist for separating out the PHC share of capital investments.^11^ Therefore, our investment guide posts for total PHC expenditure per capita are underestimated, because current expenditure is limited to recurrent costs only. Given the important role of investment in health infrastructure and equipment to improve geographical accessibility and quality of care, we advocate for future measures to include more complete reporting on capital expenditure for PHC.

### Projecting additional financing for PHC

We generate scenarios for expanding current health expenditure across all LMICs, considering three possible scenarios for growth in spending: business as usual (following the historical trend of each country); progress of 1% (achieving a 1% point increase in current health expenditure as a percentage of GDP), and an ambitious 2% (achieving a 2% point increase in current health expenditure as a percentage of GDP). To assess the potential financing gap, we calculated incremental costs and compared them with projected health spending and the share these costs would have of overall government expenditure and GDP in each country (appendix pp 37–42).

### Role of the funding source

The funder of the study had no role in study design, data collection, data analysis, data interpretation, or writing of the report. The corresponding author had full access to all the data in the study and had final responsibility for the decision to submit for publication.

## Results

**Figure 1:**
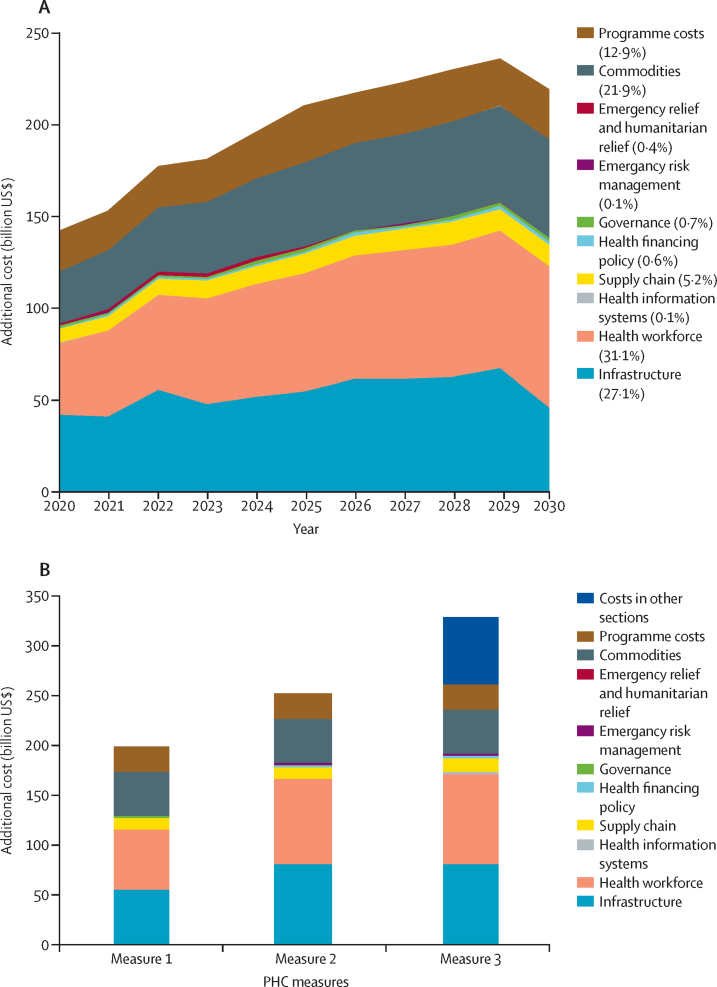
Additional investment needs for PHC (67 countries) (A) Measure 1 by year and component. (B) Additional investment needs for three measures of PHC, average 2020–30, by component. PHC=primary health care.

Estimated additional investment needs for PHC range from $200 billion for measure 1 to $253 billion for measure 2 and $328 billion for measure 3 per year from 2020 to 2030 to expand service delivery to meet SDG health targets (table 3, figure 1; appendix p 46). To provide a first set of preventive interventions and outpatient services (measure 1), an additional $2215 billion is needed over the period 2020–30, with health workforce accounting for 31·1% and infrastructure for 27·1% of costs (figure 1). Five countries (China, India, Indonesia, Nigeria, and Pakistan) account for 49·1% of costs (appendix pp 47–48). An additional $32 is needed per capita across LMICs for capital and recurrent investments, of which $28 are recurrent (table 3). For measure 1, per-capita needs are greatest in low-income countries, where recurrent spending on PHC per capita needs to increase from $25 to $65 (additional $40) with an additional $8 per capita for capital investment.

**Table 3:** Investment guide posts for PHC (measure 1), across income groups

		**Year**	**Low-income countries**	**Lower-middle-income countries**	**Upper-middle-income countries**	**All 67 countries**
**Costs**						
Total additional cost (recurrent and capital; billion US$)		Sum 2020–30	396	960	858	2215
Annual additional cost (billion US$)						
	Recurrent and capital	Average 2020–30	36	87	78	200
	Recurrent only	Average 2020-30	30	77	72	179
Current health expenditure per capita		2016	36	84	513	252
Current per capita PHC expenditure (recurrent only)*		2016	25	34	304	62
Additional PHC per capita cost (US$)						
	Recurrent and capital	Average 2020–30	48	29	32	32
	Recurrent only	Average 2020–30	40	25	29	28
Total recurrent cost per capita for PHC (current per capita PHC expenditure plus additional PHC per capita cost; recurrent only; US$)		2030	65	59	334	90
**Health workers**						
Total number of health workers, per 1000 population		Latest year available	1·4	4·6	7·9	5·6
Health workers needed for PHC services, per 1000 population		2030	5·9	6·0	8·1	6·7
**Outpatient visits**						
Total outpatient visits for modelled PHC interventions, per year, per capita		2030	5·7	4·0	8·7	6·0
Incremental outpatient visits for modelled PHC interventions, per year, per capita		2030	4·0	2·3	3·5	3·0
**Health impact**						
Total number of deaths averted because of PHC (millions)		Total 2020–30	16·2	30·6	13·5	60·1
Gains in life expectancy at birth (years)		2030 compared with 2015 baseline	6·7	4·0	2·3	3·7

Because of rounding, numbers might not add up. PHC=primary health care. All numbers are in US$ (2014) unless otherwise indicated. All per-capita numbers are population weighted.

* Using measure defined as option 5 within Van de Maele and colleagues' study.^3^

Investment guide posts indicate that the health workforce would need to increase from 5·6 per 1000 population to 6·7 per 1000 population by 2030, with an average of 5·9 outpatient visits for PHC per capita per year. Estimates for workforce and visits differ across income groups because of differing starting points (existing numbers of health workers and patient contacts are higher in middle-income countries), to which we add the required numbers of workers and visits on the basis of country-specific epidemiology and coverage targets (ie, a high untreated non-communicable disease [NCD] prevalence in many middle-income countries increases the need for outpatient visits and workforce). Examining the incremental outpatient visits for modelled PHC interventions shows that the highest additional need is in low-income countries (4·0 per year).

**Figure 2:**
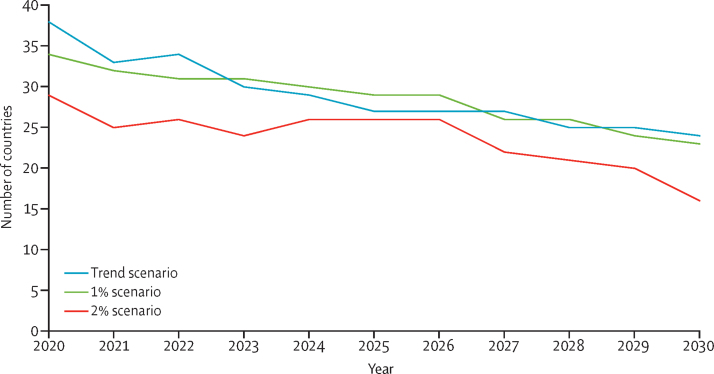
Low-income and middle-income countries with additional financing need even after increasing allocation towards primary health care (ie, countries with a gap between primary health care additional costs and projected additional finances)

Our projection model for financing indicates that if current trends continue, health expenditure across the 67 countries would increase from an average of 5·6% of GDP in 2016 to reach 6·1% of GDP in 2020 and 6·6% of GDP in 2030. However, by 2030, incremental PHC costs for measure 1 would on average require 3·3% of projected GDP (median 1·7% range 0·1–20·2; appendix pp 40–41). In a business-as-usual scenario, the number of countries with funding gaps would be 25 of 67 in 2030 (figure 2). If funding for PHC was increased by 1–2% of GDP across all countries, as few as 16 countries would see a funding gap by 2030.

**Figure 3:**
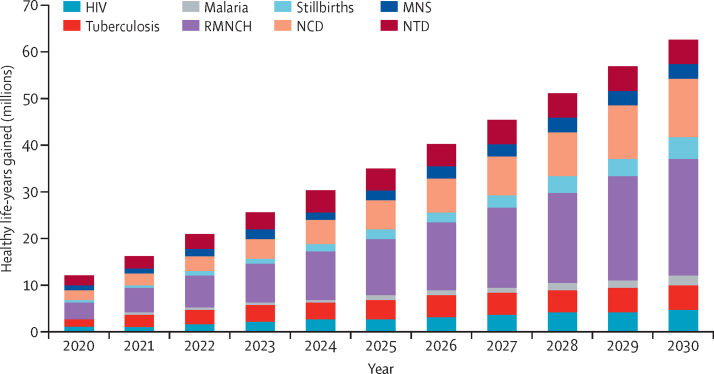
Healthy life-years gained by investments in primary health care (measure 1), by disease area (2020–30) MNS=mental, neurological, and substance use disorders. NCD=non-communicable disease. NTD=neglected tropical disease. RMNCH=reproductive, maternal, neonatal, and child health.

Increasing coverage of PHC interventions in measure 1 during 2020–30 would avert an estimated 60·1 million deaths. Inclusion of skilled care at birth (ie, measure 2) allows us to report additional maternal (1·3 million for measure 1 *vs* 1·5 million for measure 2 and measure 3) and neonatal (9·7 million for measure 1 *vs* 11·1 million for measure 2 and measure 3) lives saved, whereas inclusion of cross-sectoral interventions (ie, measure 3) within our model increases the projected number of post-neonatal deaths averted (7·7 million for measure 1 and measure 2 *vs* 8·4 million for measure 3; table 4). With measure 1, life expectancy would increase by an average of 6·7 years in low-income countries, 4·0 years in lower-middle-income countries and 2·3 years in upper-middle-income countries, using population-weighted averages (table 3). As well as increasing life expectancy, the overall health of the population will also improve, with 396 million additional healthy years of life during 2020–30 (figure 3). Most gains from PHC (for measure 1) fall within reproductive, maternal, neonatal, and child health. About three-quarters of averted stillbirths, neonatal deaths, and maternal deaths from PHC interventions are estimated to be derived from expanding access to family planning, thus reducing unplanned pregnancies and related mortality (appendix p 30). Significant gains stand to also be made for NCDs. Gains would be even greater for measure 2 and measure 3 (data not shown).

**Table 4:** Millions of deaths averted through three measures of primary health care; modelled outcomes for 67 low-income and middle-income countries, sum 2020–30

	**Measure 1**	**Measure 2**	**Measure 3**	2017 SDG price tag estimate*
Total deaths averted	60·1	63·4	64·2	89
Proportion of SDG price tag estimate	68%	71%	72%	**..**
Stillbirths	7·9	9·8	9·8	10·7
Neonatal deaths (0–1 month)	9·7	11·1	11·1	17·8
Post-neonatal deaths (1–59 months)	7·7	7·7	8·4	19·5
Under-5 deaths (neonatal and post-neonatal)	17·5	18·8	19·5	37·3
Maternal deaths	1·3	1·5	1·5	2·0
Deaths from cancer	1·9	1·9	1·9	4·1
Deaths from non-communicable diseases (four causes)	11·9	11·9	11·9	14·9
Deaths from tuberculosis	10·6	10·6	10·6	10·6
Deaths from HIV	9·0	9·0	9·0	9·0

Data are millions of deaths averted. Because of rounding, numbers might not add up. PHC=primary health care. SDG=Sustainable Development Goal.

* Results for years 2020–30 only; the original 2017 analysis presented outcomes for 2016–30.

## Discussion

We have presented a range of measures for PHC investment and estimated associated resource needs. According to our model, advancing a core set of PHC interventions in the health sector (measure 1) would require an additional $200 billion per year between 2020 and 2030 in LMICs. Expanding PHC towards a greater scope as outlined in the Declaration of Astana (ie, measure 3) would require at least $328 billion per year—and probably more.

Explicit intervention-based and input-based operational interpretations of PHC can help inform the investment agenda in LMICs. Others have previously investigated tiered models for PHC expenditure.^20^ Our approach does not imply any successive ordering of interventions because many interventions are included under measure 2 and measure 3 that bring significant health benefits and are highly cost-effective.

Countries might find that one or more measures respond more closely to their policy context. During the validation process, although country participants expressed support for having multiple measures, most global experts supported measure 1 for universal assessment, with the main reason being that measure 1 is consistent with the current PHC expenditure methods.^3^ This consistency allows a country to establish investment costs and to compare this against current expenditure. Although measures 2 and 3 are aligned with a broader interpretation of PHC in the 21st century,^12^ the absence of agreed methods for reporting expenditure for these measures limits such a comparison of projected costs and reported current spending. Nevertheless, policy should not be limited by what can be measured, and forecasted investment needs for the full scope of PHC will be essential in all countries. Although our analysis is limited by insufficient models for projecting many cross-sectoral investments in measure 3 that go beyond the scope of the original published SDG price tag analysis, within the current global programme of work,^21^ WHO is supporting the development of models to address additional PHC interventions and cross-sectoral issues to broaden future analytical scope.

Our estimates for the health SDG targets and for the subset of PHC compare in magnitude with global costs for other sectors, such as education (SDG 4), with costs estimated at 8·5% of GDP for LMICs.^22^ Although our totals, and relative additional per capita needs, give an overall impression of what remains to be done, resource needs vary across countries, depending on current and future health burdens, and the strength and structure of health systems. What is clear is that major investments need to go into system strengthening, with health workforce and infrastructure development jointly accounting for 53–66% of additional costs within the three PHC measures (appendix p 49).

The results provide evidence on likely cost drivers for PHC and shed light on the premise of affordability across countries. Expanding PHC for all requires an adequate workforce that is well motivated, well resourced, and available where needed, with accessible infrastructure and functioning equipment. We show how health workforces need to increase from 5·6 per 1000 population to 6·7 per 1000 population to support basic functioning PHC systems (measure 1). In low-income countries, this would require an ambitious four-fold increase from a current level of 1·4 health workers per 1000 to 5·9 per 1000. Strengthening health systems and scaling up services under measure 1 would require on average an additional 3·3% of GDP for PHC by 2030. In terms of financing, in a business-as-usual scenario, projected current health expenditure across the 67 countries would increase from an average of 6·1% of GDP in 2020 to 6·6% of GDP in 2030. However, countries with the smallest GDP require the largest incremental investments. Even with a 1% GDP increased allocation towards PHC, almost 25 countries would not reach the modelled benchmarks, which is unfortunate because PHC can be a remarkably efficient and highly equitable investment.^23^ Our analysis indicates that notable health gains can be achieved by investing in PHC, with up to 6·7 years gained in life expectancy in just an 11-year timeframe. For an extra $32 per capita, these health improvements have substantial value in themselves, but will also result in improved economic productivity and overall human capital.^24^ Furthermore, research shows that PHC is associated with more appropriate, effective, and less costly care.^23^ Thus, in theory, resource savings could potentially arise if countries reorient their systems towards PHC (eg, shifting services from tertiary or inpatient care to primary level or outpatient care). Our model does not take into account such shifts, because this kind of contextualised analysis would require in-depth country studies.

The impact modelling confirms the catalytic role that PHC can play for the UHC and SDG agenda. Most interventions within the SDG price tag were classified as PHC, which is consistent with Watkins and colleagues,^9^ who classified 198 of 218 essential UHC interventions as PHC. Almost 75% of the health gain previously estimated as part of the SDG agenda^10^ can be achieved through investing in PHC interventions under measure 1, with the caveat that many high-burden diseases, such as hepatitis, neurological and musculoskeletal disorders, and most cancers were not included in the impact analysis for the original SDG price tag on which the PHC-specific analysis draws. Subsequent work to expand the scope has since been done or is ongoing,^25^ and can be incorporated into future updates of PHC resource needs. Measures 2 and 3 would bring substantially larger gains. However, the current projection model is limited in terms of cross-sectoral interventions and does not calculate deaths averted from investing in other sectors (aside from water and sanitation).

Importantly, even the narrower measure 1 includes comprehensive services across disease burden and life course needs as relevant to the SDGs; this differentiates the current service-oriented approach from previous models of selective PHC, which made some significant health gains, but fell short of the vision of PHC in the Declaration of Alma-Ata.

The PHC investment guide posts were derived from a specific set of interventions within a pre-existing model,^10^ and as such do not cover the full scope of interventions defined as PHC across all country contexts. For example, the 2017 SDG projections do not include interventions for rehabilitative care or long-term home-based care. Additionally, common conditions for care seeking in LMICs include musculoskeletal complaints and endocrine, digestive system, and skin complaints,^26^ which are not explicit within our model. WHO is engaging in work to create a repository for UHC interventions, and this forthcoming work will serve to improve standardisation of information on WHO-recommended interventions, further advancing discussions around PHC. The boundaries within our model suggest that our guide posts are conservative estimates. Our model generates an average of 5·9 annual outpatient visits whereas the reported number of outpatient contacts per person per year in EU countries is 7·6.^27^ Similarly, our estimated PHC commodity costs per capita includes only a subset of interventions and assumes the use of low-cost generic medicines, whereas in reality, budget requests for medicines might be much larger. Moreover, we have estimated the investment needs from the supply side. Once functional capacities are in place, the quality of care delivered needs careful attention, including clinical effectiveness, comprehensiveness of care, and interpersonal quality of care.^1^, ^28^

Another weakness is our assumption that baselines changed little between 2015 and 2019, which might underestimate the baseline, and thus overestimate incremental resource needs. To address this, during the country consultation process we collected the most recent data for health infrastructure, health workforce, current population service coverage, and other important parameters, for inclusion in the model.

Despite the above challenges, the proposed PHC measures and investment guide posts are a conceptual framework for advancing discussions around investments in PHC. Global advocates for PHC can add more specificity to arguments for investment but in doing so should recognise that the guide posts are average estimates across countries, and as such should not be taken as absolute benchmarks for spending or health system maturity at the country level. Country policy makers can apply existing models to a locally defined PHC package and estimate resource needs. Civil society organisations can push for transparent budget allocation and benefit packages for PHC. The global community should strengthen existing evidence on recurrent and capital expenditures on PHC, advocate for targeted donor support to countries most in need, and support countries to prioritise within limited budgets.

Through our development of PHC investment guide posts we present an approach for measuring and projecting PHC investment needs in the context of advancing the UHC agenda towards the health-related SDGs. All countries should identify their own locally relevant policies for PHC, establish priority investments and associated reforms, and assess the costs and budgetary implications of these. In doing so, our approach provides a reference. Recommendations for future work include expanded models for cross-sectoral analysis, as well as projections for a more comprehensive package of health services.

## References

[1] Dugani S, Veillard J, Evans TG (2018). Quality primary health care will drive the realization of universal health coverage. CMAJ.

[2] Kluge H, Kelley E, Swaminathan S (2018). After Astana: building the economic case for increased investment in primary health care. Lancet.

[3] Van de Maele N, Xu K, Soucat A, Fleisher L, Aranguren M, Wang H (2019). Measuring primary healthcare expenditure in low-income and lower middle-income countries. BMJ Glob Health.

[4] WHO, UNICEF (Oct 25–26, 2018). Declaration of Astana. Global Conference on Primary Health Care: from Alma-Ata towards universal health coverage and the Sustainable Development Goals. Astana, Kazakhstan. https://www.who.int/docs/default-source/primary-health/declaration/gcphc-declaration.pdf.

[5] Patel M (1986). An economic evaluation of “Health for All”. Health Policy Plan.

[6] Walsh JA, Warren KS (1979). Selective primary health care: an interim strategy for disease control in developing countries. N Engl J Med.

[7] Veillard J, Cowling K, Bitton A (2017). Better measurement for performance improvement in low- and middle-income countries: the primary health care performance initiative (PHCPI) experience of conceptual framework development and indicator selection. Milbank Q.

[8] Schmidt-Traub G (Nov 12, 2015). Investment needs to achieve the Sustainable Development Goals: understanding the billions and trillions. http://unsdsn.org/wp-content/uploads/2015/09/151112-SDG-Financing-Needs.pdf.

[9] Watkins DA, Yamey G, Schäferhoff M (2018). Alma-Ata at 40 years: reflections from the *Lancet* Commission on Investing in Health. Lancet.

[10] Stenberg K, Hanssen O, Edejer TT (2017). Financing transformative health systems towards achievement of the health Sustainable Development Goals: a model for projected resource needs in 67 low-income and middle-income countries. Lancet Glob Health.

[11] Baltussen R, Jansen MP, Bijlmakers L, Tromp N, Yamin AE, Norheim OF (2017). Progressive realisation of universal health coverage: what are the required processes and evidence?. BMJ Glob Health.

[12] WHO (2018). A vision for primary health care in the 21st century: towards universal health coverage and the Sustainable Development Goals. https://www.who.int/docs/default-source/primary-health/vision.pdf?sfvrsn=c3119034_2&ua=1.

[13] Organisation for Economic Co-operation and Development, Eurostat, WHO (2011). A system of health accounts. https://www.oecd.org/publications/a-system-of-health-accounts-2011-9789264270985-en.htm.

[14] WHO (2005). International health regulations. https://www.who.int/ihr/9789241596664/en/.

[15] Prüss-Ustün A, Wolf J, Corvalán C, Neville T, Bos R, Neira M (2017). Diseases due to unhealthy environments: an updated estimate of the global burden of disease attributable to environmental determinants of health. J Public Health.

[16] GBD 2017 SDG Collaborators (2018). Measuring progress from 1990 to 2017 and projecting attainment to 2030 of the health-related Sustainable Development Goals for 195 countries and territories: a systematic analysis for the Global Burden of Disease Study 2017. Lancet.

[17] WHO (2014). WHO methods for life expectancy and healthy life expectancy. https://www.who.int/healthinfo/statistics/LT_method_1990_2012.pdf.

[18] Stop TB partnership The global plan to end TB: the paradigm shift 2016–2020. http://www.stoptb.org/global/plan/plan2/.

[19] De Vlas SJ, Stolk WA, le Rutte EA (2016). Concerted efforts to control or eliminate neglected tropical diseases: how much health will be gained?. PLoS Negl Trop Dis.

[20] Baillieu R, Kidd M, Phillips R (2019). The primary care spend model: a systems approach to measuring investment in primary care. BMJ Global Health.

[21] WHO (2019). Thirteenth general programme of work 2019–2023. https://apps.who.int/iris/bitstream/handle/10665/324775/WHO-PRP-18.1-eng.pdf.

[22] The International Commission on Financing Global Education Opportunity (2016). The learning generation: investing in education for a changing world. https://report.educationcommission.org/report/.

[23] WHO (2018). Building the economic case for primary health care: a scoping review. https://www.who.int/docs/default-source/primary-health-care-conference/phc---economic-case.pdf?sfvrsn=8d0105b8_2.

[24] Jamison DT, Summers LH, Alleyne G (2013). Global health 2035: a world converging within a generation. Lancet.

[25] Tordrup D, Hutin Y, Stenberg K (2019). Additional resource needs for viral hepatitis elimination through universal health coverage: projections in 67 low-income and middle-income countries, 2016–30. Lancet Glob Health.

[26] Salvi S, Apte K, Madas S (2015). Symptoms and medical conditions in 204 912 patients visiting primary health-care practitioners in India: a 1-day point prevalence study (the POSEIDON study). Lancet Glob Health.

[27] WHO European health information gateway. Outpatient contacts per person per year. https://gateway.euro.who.int/en/indicators/hfa_543-6300-outpatient-contacts-per-person-per-year/.

[28] Kruk ME, Gage AD, Arsenault C (2018). High-quality health systems in the Sustainable Development Goals era: time for a revolution. Lancet Glob Health.

