# Supplementary material for: Guide posts for investment in primary health care and projected resource needs in 67 low-income and middle-income countries: a modelling study
Source: Lancet Glob Health. 2019 Sep 26;7(11):e1500–10. doi: 10.1016/S2214-109X(19)30416-4 (PMC7024989; doi:10.1016/S2214-109X(19)30416-4)
Supplement: Supplementary appendix [file mmc1.pdf]

THE LANCET
Global Health

Supplementary appendix

This appendix formed part of the original submission and has been peer reviewed. We post it as supplied by the authors.

Supplement to: Stenberg K, Hanssen O, Bertram M, et al. Guide posts for investment in primary health care and projected resource needs in 67 low-income and middle-income countries: a modelling study. *Lancet Glob Health* 2019; published online Sept 26. http://dx.doi.org/10.1016/S2214-109X(19)30416-4.

Guide posts for investment in primary health care and projected resource needs in 67 low-income and middle-income countries: a modelling study

Karin Stenberg, Odd Hanssen, Melanie Bertram, Callum Brindley, Andreia Meshreky, Shannon Barkley, Tessa Tan-Torres Edejer

This document provides supplementary information to the main paper.[1] It has eight sections:

Section 1: Model parameters forthe cost and impact analysis
Section 2: Identifying PHC interventions and their related cost
Section 3: Estimating health system investments for PHC
Section 4: Methods for projecting health impact
Section 5: Constructing Investment guide posts
Section 6: Methods for estimating available financing
Section 7: Consultation and review process
Section 8: Additional results tables and figures

[1] For readers wishing additional detail to what is outlined within this document, please contact the corresponding author Karin Stenberg (stenbergk@who.int; or whochoice@who.int)

Section 1. Model parameters for the cost and impact analysis

Our analysis draws upon the WHO cost projections for reaching the health SDG targets, published in 2017 (Stenberg et al, 2017). The overall parameters for analysis are therefore the same. Here we give a general overview. For additional detail readers are advised to refer to the Stenberg et al (2017) paper.

1.1 Country selection

While the SDGs concern all countries, our model includes only low and middle-income countries, as these are faced with the greatest challenges in terms of health burden and mobilisation and effective use of resources. The selection of countries was performed in March 2016. At the time, using the World Bank income classification approach (Atlas method),[2] the 20 most populous lower middle-income countries and the 20 most populous upper middle-income countries were included, as were all low-income countries. When excluding 4 countries lacking GDP data[3] we are left with a total of 67 countries, in size representing 95% of the total population in low and middle-income countries, including a set of the most vulnerable conflict-affected and fragile contexts (Table S1).

Table S1. List of countries included in the analysis

Country	Income Group* (2019)	WHO epidemiological region	Population in millions (2019)
Afghanistan	LIC	EMRD	38.0
Algeria	UMIC	AFRD	43.1
Angola	UMIC	AFRD	31.8
Azerbaijan	UMIC	EURB	10.0
Bangladesh	LMIC	SEARD	163.0
Benin	LIC	AFRD	11.8
Brazil	UMIC	AMRB	211.0
Burkina Faso	LIC	AFRD	20.3
Burundi	LIC	AFRE	11.5
Cambodia	**LMIC**	WPRB	16.5
Cameroon	LMIC	AFRD	25.9
Central African Republic	LIC	AFRE	4.7
Chad	LIC	AFRD	15.9
China	UMIC	WPRB	1,433
Colombia	UMIC	AMRB	50.3
Comoros	**LMIC**	AFRD	0.9
Côte d'Ivoire	LMIC	AFRE	25.7

[2] https://datahelpdesk.worldbank.org/knowledgebase/articles/906519-world-bank-country-and-lending-groups

[3] The four low and middle-income countries for which we could not access GDP data at the time of the 2017 SDG analysis were Cuba, DPR Korea, Somalia and Syria.

Democratic Republic of the Congo	LIC	AFRE	86.8
Dominican Republic	UMIC	AMRB	10.7
Ecuador	UMIC	AMRD	17.4
Egypt	LMIC	EMRD	100.4
Eritrea	LIC	AFRE	3.5
Ethiopia	LIC	AFRE	112.1
Gambia	LIC	AFRD	2.3
Ghana	LMIC	AFRD	30.4
Guinea	LIC	AFRD	12.8
Guinea-Bissau	LIC	AFRD	1.9
Haiti	LIC	AMRD	11.3
India	LMIC	SEARD	1366.4
Indonesia	LMIC	SEARB	270.6
Iran (Islamic Republic of)	UMIC	EMRB	82.9
Iraq	UMIC	EMRD	39.3
Kazakhstan	UMIC	EURC	18.6
Kenya	LMIC	AFRE	52.6
Liberia	LIC	AFRD	4.9
Madagascar	LIC	AFRD	27.0
Malawi	LIC	AFRE	18.6
Malaysia	UMIC	WPRB	31.9
Mali	LIC	AFRD	19.7
Mexico	UMIC	AMRB	127.6
Morocco	LMIC	EMRD	36.5
Mozambique	LIC	AFRE	30.4
Myanmar	LMIC	SEARD	54.0
Nepal	LIC	SEARD	28.6
Niger	LIC	AFRD	23.3
Nigeria	LMIC	AFRD	201.0
Pakistan	LMIC	EMRD	216.6
Peru	UMIC	AMRD	32.5
Philippines	LMIC	WPRB	108.1
Romania	UMIC	EURB	19.4
Rwanda	LIC	AFRE	12.6
Sierra Leone	LIC	AFRD	7.8
South Africa	UMIC	AFRE	58.6
South Sudan	LIC	AFRD	11.1
Sri Lanka	LMIC	SEARB	21.3
Sudan	LMIC	EMRD	42.8
United Republic of Tanzania	LIC	AFRE	58.0
Thailand	UMIC	SEARD	69.6
Togo	LIC	AFRD	8.1
Tunisia	LMIC	EMRD	11.7
Turkey	UMIC	EURB	83.4
Uganda	LIC	AFRE	44.3
Ukraine	LMIC	EURC	44.0

Uzbekistan	LMIC	EURB	33.0
Viet Nam	LMIC	WPRB	96.5
Yemen	LMIC	EMRD	29.2
Zimbabwe	LMIC	AFRE	14.6

* Classification on July 1, 2019, World Bank Atlas method.
** United Nations, Department of Economic and Social Affairs, Population Division, World Population Prospects: The 2019 Revision, New York, 2019

1.2. Country groups and pathways towards Universal Health Coverage

Our analysis maintains the approach used in the 2017 SDG projections, which classified countries into five categories. The main purpose of the classification is to inform the modelled timing and duration of strategic investments. Countries belonging to lower level groups (Conflict, Vulnerable, Health System type 1) are assumed to be unable to scale-up as rapidly as countries belonging to higher level groups (Health System type 2 and 3), due to the more limited absorptive capacity in their systems,[4] and are also assumed to not be able to reach the same level targets by 2030 in terms of population service coverage.

Table S2. Country groups considered for the analysis

Type	Description	Criteria<br>(a) Conflict/fragility<br>(b) Resource availability[5]<br>o GNI/Capita in PPP<br>o GDP/Capita in PPP<br>(c) Service delivery readiness:<br>o HR density<br>(d) Current service delivery performance, as measured by skilled birth attendance coverage (SBA)
**Conflict-affected states (C)**	Countries with an internal or external conflict which considerably limits the state's ability to provide health services	• More than 10% of the population is affected by conflict (criteria a).
**Vulnerable countries (V)**	Countries with structural vulnerabilities, ranging from localized conflicts, a weak state apparatus, presence of external actors such as international humanitarian response structures, or recent health crisis, which limit the state`s ability to provide health services	Countries with **vulnerable systems** that have one or more of the following characteristics:<br>• Recent health system crisis (criteria a)<br>• High score on the international Fragility Index[6] (criteria a)
**Health System category 1 (HS1)**	Countries with poor performance across health system functions. These countries require an engineering of their health system in order to build the foundations of strong health system institutions, and will thus require significant investments across the health system.	Countries have limited resources and low coverage of care.<br>• GNI (PPP) / GDP (PPP) per capita falls under 2,500 (b), AND<br>• Less than 2.28 health workers per 10,000 population (c), OR<br>• SBA<90% (criteria d)

[4] One of the main factors for absorptive capacity is the available health workforce which effectively sets the production frontier. Other criteria include conflict/fragility, governance, and past performance on public expenditure management.
[5] GDP/Capita PPP used when GNI/Capita PPP is unavailable. PPP = Purchasing Power Parity –adjusted dollars.
[6] Countries with a combined score of more than 43.5 out of 50, based on scores for five key components of the Fragility Index developed by the Fund For Peace. The five components are: demographic pressures, poverty and economic decline, limits to the provision of public services, inexistence of a security apparatus, and presence of external intervention.

**Health System category 2 (HS2)**	Countries have invested in the foundations of health systems but institutional performance is poor and there are challenges related to health system efficiency and access. There is scope for rapid health system scale-up to improve performance and move towards greater domestic financing sustainability.<br><br>This includes countries that:<br>• have limited resources but are performing well in terms of SBA coverage<br>• have fewer limitations on economic resources but face challenges with respect to health worker density<br>• have fewer limitations on economic resources but are doing less well on service coverage	Countries with a combination of criteria:<br><br>• Countries that are resource constrained (GNI-PPP per capita <2,500) but perform well on a representative indicator for complex care (SBA>90%), signalling service delivery readiness that allows for quick scale up for public service coverage should resources be made available.<br><br>• Countries that are less resource constrained (GNI-PPP per capita >2,500) but where key health workforce availability is limited (HRH <2.28) , OR countries exceed the health workforce 2.28 benchmark but are doing less well on service coverage and delivery of complex services (SBA<90 %).
**Health System category 3 (HS3)**	Countries with mature health systems but in which there is an ongoing need to support health system transformation and reorient models of care to address emerging challenges and existing inequities.	This category includes:<br><br>• Countries with relatively high resource availability[7] defined as a GNI-PPP greater than 5,000, and high levels of delivery of complex care, defined as greater than 90% coverage of skilled birth attendance (criteria b, d).<br>• Countries with high resource availability defined as a GNI-PPP greater than 10,000 per capita (criteria b).

Note: this table is reproduced from Stenberg et al, 2017.

1.3 Scale-up scenarios

While the 2017 SDG projections included two scenarios (progress, and ambitious), here we consider only the ambitious scenario. This scenario describes an expansion of the full package of services towards 95% coverage for most interventions and most country categories, albeit at different speeds. It implies strengthening the foundations and institutions within health systems to enable these to support models of care that provide responsive, quality health services. It entails addressing six essential gaps by modelling investments towards attainment of benchmarks within each respective health system building block (health workforce, infrastructure, supply chain, health information systems, governance and health financing). Moreover, we model costs for emergency risk management.

1.4 Ingredients-based bottom-up costing

The general approach is an ingredients-based costing (Quantities x Prices). Within each area, we specify the inputs required to carry out activities in order to attain the benchmarks. Inputs are defined relative to total population, population density, or to other appropriate denominators such as number of districts or the projected number of health facilities per country and year. Prices are country-specific, where possible.

The non-use of unit costs implies that economies of scale (in terms of decreasing and/or increasing unit costs) is not taken into account. Instead, we consider that in certain settings, such as more sparely populated rural settings, there may be a need for more fixed resources for smaller populations than in urban settings, and as such, the implicit cost per capita is higher in most rural settings than in urban ones. A typical example is the health workforce and infrastructure modelling, where our model assumes a need for higher density of infrastructure and health workers in rural areas than in urban areas. Other than the rural-urban dimension, we do not specifically include costs for under-served groups unless specifically targeted by the interventions (for example interventions focused on men who have sex with men).

[7] More than 10,000 GNI PPP/capita, or 10,000 GDP PPP/capita when data on GNI PPP/capita unavailable.

For service delivery costs, each intervention is associated with specific inputs and prices. Cost projections are needs-based, taking into account country-specific epidemiology and coverage trajectories. This differs significantly from an approach which would project an increase in average per capita utilization visits and associated costs. A needs-based approach allows us to identify which interventions drive the costs, and to model the impact of preventive interventions on the need for curative care.

1.5 Price databases

Within our model we apply prices sourced from publicly available references and databases. Where possible, prices are differentiated by country. As a general rule, price assumptions for drugs and commodities refer to generic drugs and the lowest (median) price in the international market. [8] The WHO-CHOICE database provides country-specific prices for both traded and non-traded goods. [9] Where additional prices were needed but were not contained in the list of previously mentioned sources, we also made use of additional data sources for prices such as construction costs, [10] vaccine prices,[11] etc.

Prices are reported in 2014 USD. When costs were drawn from other pre-existing publications (such as NTDs), we adjusted the costs to 2014 USD via the same methodology.

Price assumptions within our model do not vary with volume nor over time. Thus, for example, there is no inbuilt consideration of volume discounts for drug purchases. Similarly, we have not modelled an increase or decrease in future prices[12] (e.g., salaries might be expected to increase with GDP growth, and prices of certain drugs or medicines may be expected to decrease over time). The reason for not modelling changes in prices over time is uncertainty. For many current medications it is likely that biosimilars will be forthcoming in the future patent landscape, however, outcomes on prices remain uncertain.

1.6 Tools

Our estimates are based on the 2017 WHO SDG price tag publication (Stenberg et al 2017). The analysis therefore draws on established tools and methods that have been peer reviewed and published. Most of the health service scale-up and related impact is modelled within the OneHealth Tool (OHT) Spectrum Version: 5.77 Beta 4. The OneHealth Tool is a software product whose development is overseen by a UN Inter Agency Working Group on costing (IAWG-COSTING) and carried out by Avenir Health.[13] OHT includes pre-populated country profiles that include demographic and epidemiological data specific to the country. OHT incorporates a variety of impact estimation models – including the Lives Saved (LiST) tool, the FamPlan model, and a number of models for non-communicable diseases, in order to project the costs and health impacts of scaling up specific interventions and activities in a given country. The tool is also pre-populated with cost assumptions around consumables, and the health workforce inputs required, per service provided. Table S3 below indicates which interventions are modelled within the OHT.

---

[8] MSH International Drug Price Indicator Guide http://erc.msh.org/mainpage.cfm?file=1.0.htm&module=DMP&language=English
[9] http://www.who.int/choice/cost-effectiveness/inputs/en/
[10] Data entracted from SPON`s construction costs handbooks, Compass International 2016 Construction Costs Yearbook, and IDB Infrastructure project internal reports.
[11] Portnoy et al (2015), costs of vaccine programs across 94 low-and middle-income countries.
[12] Traztuzumab for treating breast cancer is an exception, where a forecasted drop in its price is taken into account.
[13] http://who.int/choice/onehealthtool/en/

Section 2: Identifying PHC interventions and their related cost

2.1. Mapping PHC interventions to the three measures

Our starting point for the interventions to be considered under PHC was the 2017 SDG price tag. In order to identify interventions as PHC, we used the functional categories from the System of Health Accounts (OECD 2011) and applied these to the list of interventions from the SDG price tag analysis.

Under M1 we include preventive care (HC 6.1- 6.5), Home-based long-term care (HC 3.4) as well as general curative outpatient care (HC.1.3.1). Under M2 we expand the measure to also include general inpatient curative care (HC.1.1.1). Within our model, M2 is particularly relevant for maternity care, as we include basic emergency obstetric care under M2 but we exclude interventions provided as part of comprehensive emergency obstetric care. Under M3 we also include multi-sectoral interventions (HCR.2.) that were part of our model. Within the current model, these are limited to water and sanitation interventions. Table S3 provides an overview of which interventions are included in the three proposed measures of PHC (denoted with an "X"), and the model approach for projecting cost and impact.

Table S3. Interventions mapped to the three measures of PHC, according to SHA classification

Number	Intervention name	Programme [1]	PHC MeasureM 1 M1	PHC MeasureM2 M2	PHC MeasureM 3M3	SHA classification [2]	Model approach (cost and impact)
**Platform 1. Policy and population wide interventions**							
1	Increase excise taxes and prices on tobacco products.	NCD	X	X	X	HC 6.5	OHT / Excel (*)
2	Implementation of plain/standardized packaging and/or large graphic health warnings on all tobacco packages	NCD	X	X	X	HC 6.1, 6.5	OHT / Excel (*)
3	Comprehensive ban of tobacco advertising, promotion and sponsorship, including cross-border advertising and on modern means of communication	NCD	X	X	X	HC 6.5	OHT / Excel (*)
4	Elimination of exposure to second-hand tobacco smoke in all indoor workplaces, public places, public transport, and in all outdoor mass-gathering places	NCD	X	X	X	HC 6.5	OHT / Excel (*)
5	Implement effective mass media campaigns that educate the public about the harms of smoking/tobacco use and second hand smoke	NCD	X	X	X	HC 6.1	OHT / Excel (*)
6	Provision of cost-covered, effective and population-wide support (including brief advice, national toll-free quit line services and mCessation)	NCD	X	X	X	HC 6.5	OHT / Excel (*)

	for tobacco cessation to all those who want to quit						
7	Hazardous alcohol use: Enforce restrictions on availability of retailed alcohol (**)	NCD	X	X	X	HC 6.5	Excel
8	Hazardous alcohol use: Enforce restrictions on alcohol advertising (**)	NCD	X	X	X	HC 6.5	Excel
9	Hazardous alcohol use: Enforce drunk driving laws (sobriety checkpoints) (**)	NCD	X	X	X	HC 6.5	Excel
10	Hazardous alcohol use: Raise taxes on alcoholic beverages (**)	NCD	X	X	X	HC 6.5	Excel
11	Physical inactivity: Implement public awareness and motivational communications for physical activity, including mass media campaign for physical activity behaviour change	NCD	X	X	X	HC 6.1	OHT / Excel (*)
12	Sodium: Surveillance	NCD	X	X	X	HC 6.5	OHT / Excel (*)
13	Sodium: Harness industry for reformulation	NCD	X	X	X	HC 6.5	OHT / Excel (*)
14	Sodium: Adopt standards: Front of pack labelling	NCD	X	X	X	HC 6.1., 6.5	OHT / Excel (*)
15	Sodium: Adopt standards: Strategies to combat misleading marketing	NCD	X	X	X	HC 6.5	OHT / Excel (*)
16	Sodium: Knowledge: Education and communication	NCD	X	X	X	HC 6.1	OHT / Excel (*)
17	Sodium: Environment: Salt reduction strategies in community-based eating spaces	NCD	X	X	X	HC 6.5	OHT / Excel (*)
18	Diet: Complete elimination of industrial trans fats through the development of legislation banning their use in the food chain	NCD	X	X	X	HC 6.5	OHT / Excel (*)
19	Mass media (HIV/AIDS)	HIV	X	X	X	HC 6.1	OHT
20	Community mobilization (HIV/AIDS)	HIV	X	X	X	HC 6.1	OHT
21	Distribution of long lasting insecticide treated bed nets	Malaria	X	X	X	HC 6.5	OHT
22	Management of diarrhoea using Oral Rehydration Salts, zinc and increased intake of fluids	RMNCH	X	X	X	HC.1.3.1	OHT
23	Use of improved water source within 30 minutes	WASH			X	HCR.2	OHT/ Excel (*)

24	Use of water connection in the home	WASH			X	HCR.2	OHT/ Excel (*)
25	Improved excreta disposal (latrine/toilet)	WASH			X	HCR.2	OHT/ Excel (*)
26	Hand washing with soap	WASH			X	HCR.2	OHT/ Excel (*)
27	Hygienic disposal of children's stools	WASH			X	HCR.2	OHT/ Excel (*)
28	Promotion of the use of clean fuels and technologies for cooking (**)	ENV			X	HCR.2	Excel
**Platform 2: Periodic outreach services**							
29	Measles vaccine	EPI	X	X	X	HC. 6.2	OHT
30	Polio vaccine	EPI	X	X	X	HC. 6.2	OHT
31	HPV vaccine	EPI	X	X	X	HC. 6.2	OHT
32	Rotavirus vaccine	EPI	X	X	X	HC. 6.2	OHT
33	Pentavalent vaccine	EPI	X	X	X	HC. 6.2	OHT
34	DPT vaccination	EPI	X	X	X	HC. 6.2	OHT
35	Hib vaccine	EPI	X	X	X	HC. 6.2	OHT
36	Hep B vaccine to prevent liver cancer	EPI	X	X	X	HC. 6.2	OHT
37	BCG vaccine	EPI	X	X	X	HC. 6.2	OHT
38	Pneumococcal vaccine	EPI	X	X	X	HC. 6.2	OHT
39	Yellow Fever vaccine (**)	EPI	X	X	X	HC. 6.2	Excel
40	Meningitis vaccine (**)	EPI	X	X	X	HC. 6.2	Excel
41	Japanese Encephlopathy Vaccine (**)	EPI	X	X	X	HC. 6.2	Excel
42	Neglected Tropical Diseases: Preventive chemotherapy (PC) including post-PC surveillance (**)	NTD	X	X	X	HC. 6.5	Excel
43	Neglected Tropical Diseases: Vector management (**)	NTD	X	X	X	HC. 6.5	Excel
44	Neglected Tropical Diseases: Disease management	NTD	X	X	X	HC 1.3.1	Excel

	including active case finding (**)						
45	Vector control for malaria	Malaria	X	X	X	HC. 6.5	Excel
46	Chemoprevention in vulnerable populations (**)	Malaria	X	X	X	HC. 6.5	Excel
47	Clean practices and immediate essential newborn care (home)	RMNCH	X	X	X	HC. 6.4	OHT
48	Family planning	RMNCH	X	X	X	HC. 6.1	OHT
49	Outreach to injecting drug users	HIV	X	X	X	HC. 6.1	OHT
50	Needle exchange for injecting drug users	HIV	X	X	X	HC. 6.1	OHT
51	Interventions focused on female sex workers	HIV	X	X	X	HC. 6.1	OHT
52	Interventions focused on men who have sex with men	HIV	X	X	X	HC. 6.1	OHT
53	Condoms for HIV/AIDS	HIV	X	X	X	HC. 6.1	OHT
54	Iodine supplementation for pregnant women and for children (**)	Nutrition	X	X	X	HC. 6.4	Excel
55	Daily iron and folic acid supplementation (pregnant women)	Nutrition	X	X	X	HC. 6.4	OHT
56	Daily Iron folic acid, postpartum, anaemic women (**)	Nutrition	X	X	X	HC. 6.4	OHT
57	Breastfeeding counselling and support	Nutrition	X	X	X	HC. 6.1	OHT
58	Complementary feeding counselling and support	Nutrition	X	X	X	HC. 6.1	OHT
59	Nurturing care counselling for early child development	RMNCH	X	X	X	HC. 6.1	Excel
60	Support for maternal depression	RMNCH	X	X	X	HC 1.3.1	Excel
61	Home fortification of food with multiple micronutrient powders (children 6-23 months)	Nutrition	X	X	X	HC. 6.4	OHT
62	Vitamin A supplementation in infants and children 6-59 months	Nutrition	X	X	X	HC. 6.4	OHT
63	Intermittent iron supplementation in children	Nutrition	X	X	X	HC. 6.4	OHT
64	Daily iron supplementation for children 6 to 23 months (where anaemia is >= 40%)	Nutrition	X	X	X	HC. 6.4	OHT

65	Management of moderate acute malnutrition (children)	Nutrition	X	X	X	HC 1.3.1	OHT
66	Feeding counselling and support for infants and young children in emergency situations (**)	Nutrition	X	X	X	HC. 6.4	OHT
67	Offer to help quit tobacco use: Brief intervention	NCD	X	X	X	HC. 6.1	OHT/Excel (*)
68	Screening and brief intervention for hazardous and harmful alcohol use	NCD	X	X	X	HC. 6.3	OHT/Excel (*)
69	Physical inactivity: Brief advice as part of routine care	NCD	X	X	X	HC. 6.1	OHT/Excel (*)
70	Basic palliative care for breast, cervical and colorectal cancer (**)	NCD/cancer	X	X	X	HC 3.4	Excel
**Platform 3: First level clinical services**							
71	Safe abortion	RMNCH	X	X	X	HC. 6.4	OHT
72	Post-abortion case management	RMNCH		X	X	HC 1.1.1	OHT
73	Ectopic case management (medical)	RMNCH				HC 1.1.2	OHT
74	Tetanus toxoid immunization (pregnant women)	RMNCH	X	X	X	HC. 6.2	OHT
75	Syphilis detection and treatment (pregnant women)	RMNCH	X	X	X	HC. 6.3	OHT
76	Basic antenatal care (4 visits)	RMNCH	X	X	X	HC. 6.4	OHT
77	Hypertensive disorder case management	RMNCH	X	X	X	HC 1.3.1	OHT
78	Management of pre-eclampsia (Magnesium sulphate)	RMNCH				HC 1.1.2	OHT
80	Labor and delivery management - normal delivery	RMNCH		X	X	HC 1.1.1	OHT
81	Active management of the 3rd stage of labour	RMNCH		X	X	HC 1.1.1	OHT
82	Management of eclampsia (Magnesium sulphate)	RMNCH				HC 1.1.2	OHT
83	Neonatal resuscitation (institutional)	RMNCH		X	X	HC 1.1.1	OHT
84	Treatment of local infections (Newborn)	RMNCH	X	X	X	HC 1.3.1	OHT
85	Kangaroo mother care	RMNCH	X	X	X	HC. 6.1	OHT

86	Feeding counselling and support for low-birth-weight infants (**)	RMNCH	X	X	X	HC. 6.1	OHT
87	Antibiotics for preterm premature rupture of membranes (pPRoM)	RMNCH		X	X	HC 1.1.1	OHT
88	Maternal Sepsis case management	RMNCH				HC 1.1.2	OHT
89	Newborn sepsis - Injectable antibiotics	RMNCH				HC 1.1.2	OHT
90	Clean postnatal practices	RMNCH	X	X	X	HC. 6.4	OHT
91	Mastitis	RMNCH	X	X	X	HC 1.3.1	OHT
92	Chlorhexidine for cord care	RMNCH	X	X	X	HC. 6.4	OHT
93	Treatment of syphilis	RMNCH	X	X	X	HC 1.3.1	Excel
94	Treatment of gonorrhoea (**)	RMNCH	X	X	X	HC 1.3.1	Excel
95	Treatment of chlamydia (**)	RMNCH	X	X	X	HC 1.3.1	Excel
96	Treatment of trichomoniasis (**)	RMNCH	X	X	X	HC 1.3.1	Excel
97	Treatment of lower abdominal pain and Pelvic Inflammatory Disease (PID) - lower abdominal pain (**)	RMNCH				HC 1.3.3	Excel
98	Treatment of urinary tract infection (UTI) (**)	RMNCH	X	X	X	HC 1.3.1	Excel
99	Vitamin A supplementation for treatment of xerophthalmia in women of reproductive age (**)	RMNCH	X	X	X	HC 1.3.1	OHT
100	Vitamin A supplementation for treatment of xerophthalmia in children (**)	RMNCH	X	X	X	HC 1.3.1	OHT
101	Pneumonia treatment (children)	RMNCH	X	X	X	HC 1.3.1	OHT
102	Antibiotics for treatment of dysentery in children	RMNCH	X	X	X	HC 1.3.1	OHT
103	Vitamin A for measles treatment (children)	RMNCH	X	X	X	HC 1.3.1	OHT
104	Intermittent preventive treatment of malaria in pregnancy (iptp)	Malaria	X	X	X	HC. 6.4	OHT
107	Malaria diagnosis and treatment (children under five)	Malaria	X	X	X	HC 1.3.1	OHT
108	Malaria diagnosis and treatment (population aged 5	Malaria	X	X	X	HC 1.3.1	OHT

	years and above, including pregnant women)						
109	TB: first line treatment	TB	X	X	X	HC 1.3.1	Excel
110	TB: second line treatment	TB				HC 1.3.3	Excel
111	Collaborative TB/HIV activities, and management of co-morbidities	TB	X	X	X	HC 1.3.1	Excel
112	TB: diagnostic	TB	X	X	X	HC 1.3.1	Excel
113	Drug substitution for injecting drug users	HIV	X	X	X	HC 1.3.1	OHT
114	Voluntary counselling and testing	HIV	X	X	X	HC. 6.1	OHT
115	Male circumcision	HIV	X	X	X	HC. 6.4	OHT
116	Prevention of mother-to-child transmission (PMTCT)	HIV	X	X	X	HC. 6.5	OHT
117	Post-exposure prophylaxis	HIV	X	X	X	HC. 6.5	OHT
118	ART (Second-Line Treatment) for adults	HIV				HC 1.3.3	OHT
119	Paediatric ART	HIV	X	X	X	HC 1.3.1	OHT
120	Cotrimoxazole for children	HIV	X	X	X	HC. 6.5	OHT
121	HIV/AIDS service package for transgender populations (**)	HIV	X	X	X	HC 1.3.1	Excel
122	HIV/AIDS service package for prisoners (**)	HIV	X	X	X	HC 1.3.1	Excel
123	Pre-exposure prophylaxis (PrEP) (**)	HIV	X	X	X	HC. 6.5	Excel
124	Intermittent iron-folic acid supplementation (menstruating women where anaemia is public health problem)	Nutrition	X	X	X	HC. 6.4	OHT
125	Intermittent iron and folic acid supplementation (non-anaemic pregnant women) (**)	Nutrition	X	X	X	HC. 6.4	OHT
126	Vitamin A supplementation in pregnant women	Nutrition	X	X	X	HC. 6.4	OHT
127	Calcium supplementation for prevention and treatment of pre-eclampsia and eclampsia	Nutrition	X	X	X	HC. 6.4	OHT
128	Nutritional care and support (HIV+ pregnant and lactating women) (**)	Nutrition	X	X	X	HC. 6.4	OHT

129	Nutritional care and support for pregnant and lactating women in emergencies	Nutrition	X	X	X	HC. 6.4	OHT
130	Intermittent FAF, postpartum, non-anemic pregnant women (**)	Nutrition	X	X	X	HC. 6.4	OHT
131	Screening for risk of CVD/diabetes	NCD	X	X	X	HC. 6.3	OHT
132	Follow-up care for those at low risk of CVD/diabetes (absolute risk: 10-20%)	NCD	X	X	X	HC. 6.1	OHT
133	Treatment for those with very high cholesterol but low absolute risk of CVD/diabetes (< 20%) OHT	NCD	X	X	X	HC 1.3.1	OHT
134	Treatment for those with high blood pressure but low absolute risk of CVD/diabetes (< 20%)	NCD	X	X	X	HC 1.3.1	OHT
135	Treatment for those with absolute risk of CVD/diabetes 20-30%	NCD	X	X	X	HC 1.3.1	OHT
136	Treatment for those with high absolute risk of CVD/diabetes (>30%)	NCD	X	X	X	HC 1.3.1	OHT
137	Treatment of cases with rheumatic heart disease (with benzathine penicillin)	NCD	X	X	X	HC 1.3.1	OHT
138	Standard glycemic control	NCD	X	X	X	HC 1.3.1	OHT
139	Intensive glycemic control	NCD				HC 1.3.3	OHT
140	Neuropathy screening and preventive foot care	NCD	X	X	X	HC. 6.3	OHT
141	Screening and Treat pre-cancerous lesions (Cervical cancer: VIA, HPV+VIA)	NCD/cancer	X	X	X	HC. 6.3	Excel
142	Colorectal Cancer screening	NCD/cancer	X	X	X	HC. 6.3	Excel
143	Post-cancer surveillance (breast, cervical, colorectal)	NCD/cancer	X	X	X	HC. 6.3	Excel
144	Extended palliative care for breast cancer for breast, cervical and colorectal cancer	NCD/cancer	X	X	X	HC 1.3.1	Excel
145	Asthma: Inhaled short acting beta agonist for intermittent asthma	NCD	X	X	X	HC 1.3.1	OHT
146	Asthma: Low dose inhaled beclometasone + short-acting beta 2-agonists (SABA)	NCD	X	X	X	HC 1.3.1	OHT
147	Asthma: High dose inhaled beclometasone + short-acting beta 2-agonists (SABA)	NCD	X	X	X	HC 1.3.1	OHT
148	Chronic obstructive pulmonary disease (COPD): Smoking cessation	NCD	X	X	X	HC 1.3.1	OHT

149	Chronic obstructive pulmonary disease (COPD): Inhaled salbutamol	NCD	X	X	X	HC 1.3.1	OHT
150	Chronic obstructive pulmonary disease (COPD): Low-dose oral theophylline	NCD	X	X	X	HC 1.3.1	OHT
151	Chronic obstructive pulmonary disease (COPD): Ipratropium inhaler	NCD	X	X	X	HC 1.3.1	OHT
152	Basic psychosocial treatment for anxiety disorders (mild cases)	MNS	X	X	X	HC 1.3.1	OHT
153	Basic psychosocial treatment and anti-depressant medication for anxiety disorders (moderate-severe cases)	MNS				HC 1.3.3	OHT
154	Basic psychosocial treatment for mild depression	MNS	X	X	X	HC 1.3.1	OHT
155	Basic psychosocial treatment and anti-depressant medication of first episode moderate-severe cases	MNS				HC 1.3.3	OHT
156	Intensive psychosocial treatment and anti-depressant medication of first episode moderate-severe cases	MNS				HC 1.3.3	OHT
157	Basic psychosocial support and anti-psychotic medication	MNS	X	X	X	HC 1.3.1	OHT
158	Intensive psychosocial support and anti-psychotic medication	MNS				HC 1.3.3	OHT
159	Basic psychosocial treatment, advice, and follow-up for bipolar disorder, plus mood-stabilizing medication	MNS	X	X	X	HC 1.3.1	OHT
160	Intensive psychosocial intervention for bipolar disorder, plus mood-stabilizing medication	MNS				HC 1.3.3	OHT
161	Basic psychosocial support, advice, and follow-up, plus anti-epileptic medication	MNS	X	X	X	HC 1.3.1	OHT
**Platform 4: Care provided at first level and above**							
162	Labor and delivery management - emergency obstetric care	RMNCH		X (BEmOC)#	X	HC 1.1.1	OHT
163	Pre-referral management of labor complications	RMNCH		X	X	HC 1.1.1	OHT
164	Management of obstructed labor	RMNCH				HC 1.1.2	OHT
165	Antenatal corticosteroids for preterm labor	RMNCH		X	X	HC 1.1.1	OHT
166	Induction of labor (beyond 41 weeks)	RMNCH		X	X	HC 1.1.1	OHT

167	Newborn sepsis - Full supportive care	RMNCH				HC 1.1.2	OHT
168	Treatment of postpartum hemorrhage	RMNCH				HC 1.1.2	OHT
169	Treatment of severe illness in children (diarrhea, pneumonia, malaria)	RMNCH				HC 1.1.2	OHT
166	Management of severe malnutrition (children)	Nutrition				HC 1.1.2	OHT
167	Retinopathy screening and photocoagulation	NCD	X	X	X	HC 1.3.1	OHT
170	Treatment of new cases of acute myocardial infarction (AMI) with aspirin	NCD	X	X	X	HC 1.3.1	OHT
171	Treatment of cases with established ischaemic heart disease (IHD) and post MI	NCD	X	X	X	HC 1.3.1	OHT
172	Treatment for those with established cerebrovascular disease and post stroke	NCD	X	X	X	HC 1.3.1	OHT
173	Mammography	NCD/cancer	X	X	X	HC 6.3	Excel
174	Cervical cancer treatment: stage 1 to stage 4	NCD/cancer				HC 1.3.3	Excel
175	Colorectal cancer treatment: stage 1 to stage 4	NCD/cancer				HC 1.3.3	Excel
176	Breast cancer treatment: stage 1 to stage 4	NCD/cancer				HC 1.3.3	Excel
177	Asthma: Theophylline + High dose inhaled beclometasone + SABA	NCD	X	X	X	HC 1.3.1	OHT
178	Asthma: Oral Prednisolone + Theophylline + High dose inhaled beclometasone + SABA	NCD	X	X	X	HC 1.3.1	OHT
179	COPD: Exacerbation treatment with antibiotics	NCD	X	X	X	HC 1.3.1	OHT
180	COPD: Exacerbation treatment with oral prednisolone	NCD	X	X	X	HC 1.3.1	OHT
181	COPD: Exacerbation treatment with oxygen	NCD	X	X	X	HC 1.3.1	OHT
182	Intensive psychosocial treatment and anti-depressant medication for anxiety disorders (moderate-severe cases)	MNS				HC 1.3.3	OHT
183	Intensive psychosocial treatment and anti-depressant medication of recurrent moderate-severe cases on an episodic basis	MNS				HC 1.3.3	OHT

184	Intensive psychosocial treatment and anti-depressant medication of recurrent moderate-severe cases on a maintenance basis	MNS				HC 1.3.3	OHT
185	Surgical and trauma care	Surgery				HC 1.1.2; HC 1.3.3.	Excel
**Additional programmatic interventions incl. activities addressing socioeconomic determinants**							
186	Cash transfers for girls in hyper-endemic countries with low rates of secondary school enrolment (**)	HIV			X	CROSS-SECTORAL	Excel
187	Cash transfer to poor women to deliver in facilities (**)	RMNCH			X	CROSS-SECTORAL	Excel
188	Programme support costs include training, monitoring, supervision, programme administration costs. (**)	ENV, EPI, HIV, NCD, Malaria, MNS, NTD, Nutrition, RMNCH, Surgery, TB				Included under HC 6	Excel

Notes to table:

1. Programme abbreviations: ENV= Environmental health; EPI = Expanded Program on Immunization, MNS = Mental Health and Substance Use; NCD = Non Communicable Disease; NTD= Neglected Tropical Diseases, OHT = OneHealth Tool, RMNCH = Reproductive, Maternal, Child and Newborn Health.
2. SHA classifications used in this table:
   HC 1.1.1 General inpatient curative care
   HC 1.1.2. Specialized inpatient curative care
   HC.1.3.1 General curative outpatient care
   HC.1.3.3 Specialised curative outpatient care
   HC 3.4 Home-based long-term care
   HC. 6.1 Information, education and counselling programmes
   HC. 6.2 Immunisation programmes
   HC. 6.3 Early disease detection programmes
   HC. 6.4 Health condition monitoring programmes
   HC. 6.5 Epidemiologic surveillance and risk and disease control programmes
   HCR.2 Health promotion with multi-sectoral approach
3. Intervention classification
   # Basic Emergency Obstetric Care only is counted as PHC under M2.
4. Model approach
   OHT = the OneHealth Tool
   (*) Health impact was projected within the OHT projections, while costs were modelled in Excel.
   (**) No health impact modelling directly associated with this intervention.

Some interventions that were originally classified under platform 4 within the 2017 SDG price tag analysis were now considered within the PHC measures 1, 2 and 3. Thus, applying the SHA classifications illustrates that some interventions commonly associated with referral level type platforms can, and should be, considered as part of PHC and be made available through close to client general outpatient care services.

We acknowledge that the list of interventions presented in table S3 does not represent a complete list of PHC interventions that a country may wish to consider delivering. There are important areas missing, including oral health, vision and hearing screening, rehabilitation, skin care, and many other conditions for which people seek care at primary health care. Moreover, interventions are not always standardised across disease areas or health programmes in terms of their granularity. To address these issues, WHO is now engaging in work to create a repository of WHO-recommended interventions to be considered for PHC and UHC. This forthcoming repository will include a broader set of interventions than what is covered in Table S3, and can hopefully inform future updates of global resource needs; as well as to provide guidance to countries on the types of interventions to consider providing. As the repository will be continuously updated to reflect the latest evidence, it will include considerations of innovative delivery mechanisms, digital health and telemedicine.

To identify costs associated directly with PHC delivery, we extracted the commodity cost for the relevant set of health interventions under each scenario. For interventions within the OneHealth Tool, the intervention-specific treatment inputs are based on WHO guidelines. For interventions modelled outside the OneHealth Tool (i.e., TB, NTDs and cancers), we extracted the PHC-related commodity and programme cost from the respective models.

Costs for activities to support programme administration and scale-up were estimated within the SDG 2017 model for each programme (maternal and child health, SRHR, immunization, nutrition, malaria, HIV/AIDS, NCDs, cancers, mental health and substance use, neglected tropical diseases, and environmental health) using the WHO-CHOICE standardised programme costs (www.who.int/choice) and using a tracer intervention approach for each programme. Here we include the programme costs in full for all three PHC measures, since each programme includes PHC interventions. These programme administration costs include costs for training health workers, monitoring and evaluation of programme performance, supervision, information campaigns and general programme management.

Details on health system costs are described in the next section.

Section 3: Estimating health system investments for PHC

This section describes the approach taken for the different health system areas, emergency preparedness and response, and specific investments in distressed and post-conflict settings.

3.1 Infrastructure and equipment

3.1.1. Context and previous resource estimates

For the additional costs for health infrastructure, we once again draw upon the WHO 2017 SDG analysis and modify it such that only PHC related costs are captured. Here, the model was developed to compare current numbers of health facilities with a set of targets based on facility densities for population catchments, as identified below.

Table S4. Categories of health facility infrastructure considered within the model

Facility	Population Catchment	Notional Staff	Size (sqm)	Years to build	Beds	Vehicles
Urban Health Center	12,000	5	230	1	X	Share of monitoring vehicle
Rural Health Center	6,000	3*	115	1	X	Share of monitoring vehicle
Urban District Hospital	100,000	50	8,000	3	100	1 ambulance
Rural District Hospital	50,000	25*	4000	3	50	1 ambulance
Provincial Hospital	1,000,00	200	40,000	5	500	2 ambulances

*Community health workers who do outreach in rural settings are not included in the category of notional staff.

The infrastructure model explicitly considers health workers and health facilities jointly, at each level of care. This entailed modelling a paired scale up of health workers and facilities. Targets take into account the health services to be provided as part of each service delivery platform, as well as projected future population growth, population density, and rural-urban migration. Within the model, we pay specific attention to the need to strengthen accessibility to health facilities in rural areas in order to respond to unmet needs of rural populations.

Costs include construction of new infrastructure, their related equipment needs, and the recurrent costs that these will accrue once operational. We also include investments for a proportion of existing facilities to be upgraded to meet “Safe Hospital” standards to be resilient to external shocks. Additionally, we model improvements in making water and power lines available in those facilities not yet connected, in order to expand quality of care and access to basic services.

Finally, within higher level facilities, we estimated costs for vehicles and related operational costs (e.g., maintenance, fuel), including the referral chain.

We modelled the scale-up over time towards density targets following a series of scale-up curves that varied across the five country typologies, as well as between the five types of facilities. Our model assumes that countries categorized as conflict and vulnerable would not be expected to start building additional facilities until they become stable enough to allow for construction activities.

Health facility construction costs were gathered from a variety of sources, including construction cost publications and primary data provided by a regional development bank[14]. Maintenance costs are assumed to be 10% of replacement costs, while capital depreciation is estimated as 3% of annualized values of capital[15]. For operating costs, facilities were assumed to use factors of per-capita annual usage of utilities, with health centers assumed to consume the electricity and water equivalent to the average used by 10 people in their homes a day. We searched planning documents from low and middle income countries for guidance on assumptions of telecommunications components and the profile of medical equipment and supplies. Costs for non-medical equipment, such as furniture, computers, backup generators and fuel, are taken from the WHO CHOICE database. The cost of a new district surveillance vehicle (prices also from the WHO CHOICE database), is assumed to be shared between the health centers within a district, and as such a share of its fuel costs its attributed to each new health center. We assume that each new district and provincial hospital would have 1 and 2 ambulances, respectively, with respective fuel and driver wage costs taken into account. Finally meeting Safe Hospital standards in new facilities is included as 4% of the basic infrastructure cost of each facility, based on PAHO-World Bank guidelines highlighted in the 2008-2009 World Disaster Reduction Campaign[16].

Moreover, we modelled the additional costs borne by the health system if health facilities that did not have access to water and electricity were connected to the utilities grid. The number of facilities without these utilities were estimated, and modelled as being connected to utilities at the same rate of scale up of health centers. The additional electricity and water costs were then added to the recurrent costs of those facilities for each country. The costs of power lines or pipes for the actual connection of facilities to utilities was not estimated.

3.1.2 Identifying infrastructure costs for PHC within the WHO 2017 SDG model

While an intuitive approach to PHC may simply suggest including the cost of primary health centers as the infrastructure costs for PHC, this is not our approach, as PHC services can be delivered across all levels of the health system. Therefore, we include all costs for building, equipping and refurbishing health centers, as well as a share of the costs of district hospitals and provincial hospitals, based on health accounts data on expenditure patterns within hospitals.

---

[14] SPON`s construction costs handbooks, Compass International 2016 Construction Costs Yearbook, IDB Infrastructure project internal reports.
[15] World Bank, 1994. *Better health in Africa: experience and lessons learned.*
[16] PAHO/World Bank (2003), *Protecting New Health Facilities from Natural Disasters: Guidelines for the Promotion of Disaster Mitigation.* Documentation for the WDR campaign, by International Strategy for Disaster Reduction, the WHO and the World Bank, can be found at: http://www.unisdr.org/2009/campaign/wdrc-2008-2009.html

Table S5. Overall health expenditure by functional category (HC), Average across low-and middle income countries (N=21), from WHO Global Health Expenditure Database

	General inpatient	General outpatient	Specialized inpatient	Specialized outpatient	Other functions (not specific to inpatient/outpatient)
District (general) hospitals	48%	33%	2%	3%	14%
Provincial hospitals	24%	3%	40%	10%	23%

Estimating Infrastructure costs for PHC M1

For MM1 we include the full costs of building, refurbishing and operating health centers. This includes costs for purchasing and maintaining equipment. Moreover we include a percentage share of the cost required to construct, refurbish and equip district hospitals (33%) and provincial hospitals (3%). The share is derived from expenditure data on non-specialized outpatient care within this type of facilities, as shown in Table S5.

Estimating Infrastructure costs for PHC M2

Under MM2, we expand the cost to consider a larger share of cost at district hospitals (81%) to account for non-specialized inpatient care. Furthermore, we also include a larger share of costs for constructing and running provincial hospitals (24%), to account for non-specialized outpatient and inpatient PHC care delivered in these facilities.

Estimating Infrastructure costs for PHC M3

For MM3 we used the same approach as for Measure 2, i.e., the costs are identical.

Reallocation of capital costs
It should be noted that for all three measures M1M2M3M1, M2, M3 we reallocated costs for 2016-2019 from the original SDG model to be incurred in years 2020-2030, in order to capture the full cost for infrastructure as estimated within the model.

3.2 Health workforce

3.2.1.Context and previous resource estimates

The 2016-2030 Global Strategy on Human Resources for Health[17] ("Global Strategy") outlines the critical role of the health workforce to achieve the Sustainable Development Goals and Universal Health Coverage. The report presented joint work by WHO and the World Bank indicating that approximately 40 million new jobs will be created in the health and social care sectors globally by 2030 but that there is a need for 18 million additional health workers, primarily in low-resource settings, to attain high and effective coverage of necessary health services. A composite index threshold of 4.45 physicians, nurses and midwives per 1000 population was used to estimate the health workforce needs and needs-based shortages for each country by 2030. The analysis was done for three cadres: Medical doctors, Nurses and midwives, and Ancillary and Other health workers[18].

[17] WHO (2016), Global strategy on human resources for health: workforce 2030.
[18] Other researchers have looked at alternative methods for modelling the health workforce needs of the future, including for other, more specific types of health workers. Future analysis for health worker needs as part of a

The Global Strategy was used as the starting point for WHO's 2017 SDG analysis. The 2017 analysis foresaw countries scaling up to attain universal health coverage following a PHC approach. As primary health care in rural areas will be more focused on outreach activities and require more community and public health workers than in urban areas, the model used within the Global Strategy was adjusted to apply a higher target density for "other cadres" in rural as compared to urban areas. The composition of stylized health teams was modelled across different types of facilities as shown in Table S6. The intent was to take into account that different service delivery platforms have different health workforce requirements.

Table S6. Stylized Health Worker teams in each facility as per WHO's 2017 SDG analysis

	Urban Health Center	Rural Health Center	Urban District Hospital	Rural District Hospital	Provincial Hospital	Total
Doctors	2 (.16)	2 (.34)	10(.1)	10 (.2)	40 (.4)	(1.2)
Nurses and midwives	5 (.42)	5 (.83)	25 (.25)	25 (.50)	125 (1.25)	(3.25)
Other health workers	3 (.25)	4+12* (.50+1.5*)	35(.35)	35+ 30* (.70+.5*)	250 (.25)	(2.14 +2*)

In order to derive the incremental cost for health workers, the model compared baseline number of workers with the population-target ratios. The current number of health workers were derived from an updated database of global health worker density, based on values in the WHO global health observatory, and updated with inputs from country representatives, where available. They were then classified according to the three categories of medical doctors, nurses and midwives, and "other cadres". Projected supply densities of health workers from 2016 to 2030 were taken from the work of the Global Strategy on Human Resources for Health. These were calculated using a linear growth rate model based on historical trends by country. Where projected supply densities showed a projected increase, but had a lower projected initial density than the observed value, the first year supply densities based on trendline results were increased to not show this drop-off relative to the latest measured health worker densities.

In the 2017 study, the projected supply of each type of health worker by year for each country was compared with the SDG threshold quantity, and when there was a gap (indicating a shortage), we modelled investments beyond and above the historical trends, successively closing the gap by 2030. Scale-up curves vary across the five country typologies, as well as between the three types of workers.

Incremental costs for the health workforce comprise the salaries of all additional health workers, incremental to baseline (2015) levels: this includes both health workers modelled as joining the labor force after 2015 based on projected linear growth trends, as well as additional outputs required to close the gap. We assume an annual attrition rate of around 3%, taking into account country-specific rates of factors that contribute to attrition, including mortality.

Country-specific health-worker salary estimates were taken from the WHO-CHOICE database. In-service training costs are included based on an assessment of a standardized investment by disease/health programme. The cost estimates were then aggregated and reduced by 10% to represent an integrated approach to continued professional development.

---

resource needs exercise should apply this approach, but as seen by its application in mostly high income countries, data requirements may present a limitation. For more detail, see: Tomblin Murphy et al, 2016, and Mackenzie, Tomblin Murphy, & Audas, 2019.

3.2.2 Identifying health workforce costs within the WHO 2017 SDG model

To identify PHC-specific health workforce costs, we adjusted the model used for the SDG analysis, and adopted a bottom-up approach. For each country, we developed three scenarios within the OneHealth Tool to match the three measures of PHC. We then extracted the total number of minutes of health workforce time required to deliver the additional PHC interventions, per cadre, per country and year from the OneHealth Tool scenarios. This provided us with the total number of minutes of time required to deliver the identified package of OHT interventions. In addition, for one set of interventions, cancer care, the total number of minutes of health workforce time required these interventions was estimated outside of the OHT, in Excel, and added to the OHT-based minutes of additional health worker time.

The number of minutes were then converted into Full Time Equivalents using assumptions around the amount of time providers spend in direct contact. [19]

We similarly extracted minutes and estimated Full Time Equivalents for the additional delivery of health services within the original 2017 SDG scenarios. This allowed us to calculate the required time to deliver PHC interventions as a share of the total time estimated for the full package of SDG price tag services analysed within the OneHealth Tool (OHT).

We thus have three measures:

- 2017 SDG total HR estimates using population density ratios. (SDGpr)
- 2017 SDG bottom -up estimates from OHT interventions (SDG bu)
- 2019 PHC bottom -up estimates from OHT interventions (PHC bu)

By definition the HR required for delivering a package of PHC interventions is a subset of the HR required to deliver the services to attain the SDGs, and as such PHCbu / SDGbu < 1. The population-density based health worker ratios used in the SDG price tag were intended to cover the necessary health workers to cover a comprehensive set of UHC services, and as such went beyond the services that were modelled within the OHT. Similarly, the OHT does not include a full set of PHC services, and therefore the bottom-up HR estimates are an underestimate. There are essential PHC services that are not being captured by the HR estimates derived from the OHT. Therefore, HR estimates for M1, M2 and M3 should be adjusted upwards to reflect this. Moreover, MM2 and MM3 include inpatient services, which are particularly limited within the OHT. M2 and M3 are therefore adjusted upwards further to take inpatient care into account. The adjustments use the same approach as adopted for infrastructure, i.e., to apply a share of costs for care provided in district and provincial hospitals.

Modelling carried out outside of the OHT for health worker time related to delivering cancer interventions is added to the bottom up estimates mentioned above, for all PHC as well as the SDG bottom up estimates. However health workforce time estimates exclude the time to deliver interventions to address neglected tropical diseases and TB.

Estimating Health Workforce costs for PHC M1

---

[19] For nurses and midwives we used assumptions consistent with a study by Westbrook JI1, Duffield C, Li L, and Creswick NJ (2011) and assumed that nurses and midwives would spend around 40% of their time in direct contact with patients. For medical doctors we assumed 15% of time spent in direct contact with patients/families as per Westbrook et al (2008). We assumed 8 hour working days, 220 days per year.

To estimate the health workforce required for delivering PHC services for measure 1, we take a proportional share of the workforce estimated to be required for reaching the health SDGs. This is estimated, for each country and year, by the following equation: PHC work force= PHCbu/SDGbu * SDGpr.

Estimating Health Workforce costs for PHC M2

Measure 2 includes an additional component of health worker time for responding to generalized inpatient care. As noted above, the inpatient care interventions in the OHT are limited, and as such we have limited information available on required HR time. Therefore, our approach was to allocate an additional share of SDGpr. The share added is set in proportion to the additional cost for hospital infrastructure added to MM2, proxied by the percentage share increase in total hospital beds per capita resulting from the construction of district hospitals.

As with M1, M2 has a bottom up quantity of health workforce, $PHCbu^{M2}$ which is divided by the SDGbu, and therefore adjusted by the population-density number used in the SDG pricetag, SDGpr. As we add additional inpatient general services for M2, which are delivered in district and provincial hospitals, we have to add a share of the workforce that are SDGpr that were not part of the resulting workforce in M1, PHCM1. In other words, we add a share the difference between the two, SDGpr-PHCM1, one piece corresponding to the additional services delivered in district hospitals that were not part of M1, and another piece that corresponds to the additional services delivered in provincial hospitals that were not part of M2. Since there is no clear way to determine which additional workers within the model are being added to which level, we use a proxy of the additional beds added to the health system to capture the additional workers being assigned to the two hospital levels. Therefore, to account for the workers needed to deliver the additional services at district hospitals, we add a proportional share of the difference above, equal to the beds added to the health system that come from district hospitals (new beds district hospitals/total new beds) adjusted by an allocation factor proportional to those outpatient generalized services that make up M2. Similarly, to account for the workers needed to deliver additional services at provincial hospitals, we add a proportional share of the difference above, but this time equal to the beds added to the health system that come from provincial hospitals (new beds in provincial hospitals/total new beds), adjusted by provincial-hospital allocation factor.

The allocation factor for district hospitals is computed as the difference between of 81% - 33%= 48%, based on the share of spending on services at district hospitals that are characterized as M2 that were not within M1, as shown in Table S5. Similarly, for provincial hospitals, this is computed as the difference between 24% -3%= 21%, based on the spending on PHC interventions that are part of M2, but not part of M1.

Estimating Health Workforce costs for PHC M3

For M3, the same approach as for M2 was used, but noting that the PHC bottom up estimate is slightly larger, reflecting the inclusion of additional health interventions.

3.3 Supply chain

3.3.1.Context and previous resource estimates

Within WHO's 2017 SDG estimate, costs were modelled based on the estimated additional volume and value of the consumables transported through the system. The approach followed the standard USAID | DELIVER PROJECT approach, to measure costs at different levels of the supply chain: central, zonal, district, and service delivery (McCord et al, 2017). Capital costs include additional warehouses and supply vehicles needed to store and transport commodities. For recurrent costs, each commodity considered had an estimated corresponding unit volume, drawing primarily from an existing product database (USAID | DELIVER PROJECT 2016). Aggregating the volume for all commodities provides the total volume of commodities that need to be managed by the supply chain per country and year. Based on several factors affecting the efficiency of the supply chain, including population density, and logistics scores from the Wold Bank Logistics Performance Index, a logistics cost fraction was estimated for each country, for each square meter of medical commodities, that had to be handled by the supply chain, that then represented the recurrent cost for each country, in each year.

Given that the cost projections for commodities are specific to a certain set of health interventions, they may underestimate the total volume of goods required for a fully functioning health system. A mark-up of 21% was therefore added to cover costs related to commodities not included in the intervention specification list, but nonetheless required in a functioning health system, such as surgical masks and disposable gloves. Furthermore, an additional 10% mark-up was added to cover buffer stock for all commodities. However, this does not include international shipping or insurance costs.

For temperature-sensitive commodities, such as vaccines, we specifically estimate costs related for the cold chain, which are determined by estimating the cold space required to store the volumes of vaccines required to be able to provide immunization services in the subsequent year, as the storage capacity needs to be in place before mobilization and supplies of vaccines can be increased.

3.3.2 Identifying supply chain costs for PHC within the WHO 2017 SDG model

PHC services are a share of the overall supply chain costs for delivering services towards reaching the health SDGs.

Estimating Supply chain costs for each measure of PHC

To derive the supply chain costs for each measure of PHC, we calculated the cost of commodities for delivering the list of PHC services defined under each measure (M1, M2, M3). We then compared this cost to the overall estimated commodity cost computed for the overall SDG model in 2017, and computed the share. We then applied this share to the overall supply chain costs modelled for the SDG package, in order to take a proportion of the costs.

This approach does not take into account the volumes of the exact commodities required to deliver PHC services, but we consider it a good enough proxy to inform global estimates.

The costs specific to cold chain were included in their entirety for all three measures.

3.4. Health Information Systems

3.4.1.Context and previous resource estimates

Within WHO's 2017 SDG estimates, costs were modelled for a series of health information system components, drawing on an existing model of scaling up Health Information Systems that is part of the OneHealth Tool. This included resources needed for setting up a financial information system, a health workforce information system, a facility-based information system, the periodic occurrence of surveys, and the reinforcement or creation of a health information systems team within

the public system, either as part of the Ministry of Health, or within a public health institute, or within a National Statistics Office. Costs included within these are costs for specialized health workers, equipment, and governance related activities.

The scale up of the health information system for facilities and the financial information system directly follows the scale up (construction) of health facilities, while the other components follow a scale up of transition through various levels of development of systems, until reaching a maximum level of development.

3.4.2 Identifying HIS costs for PHC within the WHO 2017 SDG model

Within the PHC definitions used for the health accounts framework, there was no explicit consideration of spending related to health information systems. Therefore, we have considered the appropriateness of each cost component and its relevance to each measure of PHC.

Estimating HIS costs for PHC M1

For Measure 1, we limit HIS costs to be included to only focus on the health facility-based information system. We include the full cost for the facility-based health information system (i.e., not a share), as it is not possible to attribute or consider only a part of the system.

Estimating HIS costs for PHC M2

When expanding the scope for M2, we include all of the cost components estimated for health information systems for the health SDG 2017 model. As stated above, these include the health facility-based information system, a health workforce information system, a financial information system, surveys with health related questions, and the creation or strengthening of a HIS team within the health sector.

Estimating HIS costs for PHC M3

The scope for M3 goes beyond the health sector. We therefore include two additional components that go beyond the financial responsibility of the health system; civil registry systems and periodic censuses.

3.5 Governance

3.5.1.Context and previous resource estimates

To estimate costs, a series of activities have been identified, originally in work of the High-level Taskforce on Innovative International Financing for Health Systems (2009).[20] These were then developed further for the 2017 WHO SDG model, which was used here.

When considering what specific set of activities or functions a government should play in operationalizing the "core functions" of health systems governance, the model is built upon a framework by Siddiqi et al (2009), which identifies ten overarching principles that are key to good governance, across three levels of assessment; national, health policy formulation, and policy implementation. Certain domains are excluded from Governance costs on the grounds that they are more appropriately covered under a different 'building block' of the health system (e.g. health information systems, human resources for health), each of which are subject to their own resource need assessments in our exercise.

[20] WHO (2009), High-level Taskforce on Innovative International Financing for Health Systems

Table S7. Resource needs for specified governance activities

Intervention area	Activity	Illustrative resource needs		
		Human resources	*Training*	*Meetings*
Strategic planning & evaluation	Health planning & evaluation	Strategy unit	Strategic planning & evaluation	National health policies (development, revision)<br>Ethical guidelines / review committees
Stakeholder collaboration	Consensus building	External relations unit	Social determinants / equity in health	Stakeholder consultation (public, private & NGO sectors)
Regulation & oversight	Audits *(operations, performance)*	Internal audit unit	Audit	
	Licensing *(facilities, drugs)*	Licensing unit<br>Drug regulatory unit		
	Contracting *(with service providers)*	Contracting unit	Regulation & contracting in health	National contracting policy<br>Framework agreements
	Self-regulation *(professions)*	Regulatory bodies		Board meetings
	Accreditation *(facilities, providers)*	Accreditation agencies		Board meetings

Note: Table developed by Dan Chisholm, WHO, drawing on Siddiqi (2009).

The model estimates quantities of human, physical, and other resources needed to implement governance related activities. The World Bank`s Country Policy and Institutional Assessment (CIPA) is used to identify current levels of health system governance by country. Resource profiles to implement the required activities were constructed for each of the six possible levels along the CPIA scale, with 6 taken as the benchmark for an ambitious scenario. Countries are modelled as improving their levels of governance over time, where the effects of activities and investments in stakeholder consultation and collaboration, regulation, and strategic planning increases a country`s governance enough to bring about an increase in the CPIA score after three years. Prices and salary data for the different components considered were taken from the WHO-CHOICE database.

3.5.2 Identifying Governance costs for PHC within the WHO 2017 SDG model

For PHC Measure 1 we included 80% of the estimated costs per country for improving governance. The source for this assumption is the approach followed by the primary health care performance initiative,[21] which is also reflected in proposed measure 5 for PHC expenditure monitoring within the health accounts framework (Vandemaele et al., 2018).

For measures M2 and M3 we included the full cost (100%) for governance-related investments included in the original SDG resource need estimates.

[21] The primary health care performance initiative. Primary Health Care Vital Signs Profiles: Detailed Methodology Note [Internet]. 2018. Available from: https://improvingphc.org/sites/default/files/VSP_Detailed_Methodology_Note.pdf

3.6 Health Financing

3.6.1.Context and previous resource estimates

Within the 2017 analysis, this component estimates the resources required to improve health financing towards achieving Universal Health Coverage through strengthening the purchasing functions of social health insurance institutions and Ministries of Health who have public service provision.[22] After an assessment of countries that have either recently started or will shortly start reforming their health financing systems, it was estimated that countries should be spending 1 to 2% more of their General Government Health Expenditure (GGHE) to strengthen the administrative sections within existing Social Health Insurance institutions and Ministries of Health, in order to achieve more effective reform of their health financing functions.

3.6.2 Identifying health financing-related costs for PHC within the WHO 2017 SDG model

For measure M1 we included 80% of the estimated costs for health financing. Similar to governance-related costs, the source for this assumption is the approach followed by the primary health care performance initiative,[23] which is also reflected in proposed measure 5 for PHC expenditure monitoring within the health accounts framework (Van de Maele et al., 2018).For M2 and M3 we included the full cost (100%).

3.7. Emergency Risk Management

3.7.1.Context and previous resource estimates

Since the Ebola crisis in western Africa, a great deal of international attention has shifted towards emergency risk management and health security. Several studies have focused on the existing gaps towards preventing and being better prepared for similar epidemics in the future, and the National Academy of Medicine[24] attempted to "make the case for investing in pandemic preparedness" and made an estimate for the necessary additional resource needs to this end, where upgrading national pandemic preparedness capabilities, following on an earlier World Bank report[25], was estimated as $3.4 billion. However, this, while including animal as well as human health investment needs, was not a systematic assessment of resource needs towards the different parts of an emergency disaster risk management system, as well as extrapolated to the globe based on a handful of countries.

As part of the resource needs for reaching the health SDGs, Stenberg et al (2017) estimated three components within Emergency Risk Management. Two were resource needs linked to continued investment needs for countries to recover from emergencies, through post conflict reconstruction and post-emergency relief. The third was a series of specific investments required for emergency preparedness and risk mitigation.

For the costs of post conflict reconstruction, estimates of the cost of repair and reconstruction of health facilities that were either damaged or severely damaged or destroyed were estimated. This uses published costs of repair, using country-specific costs where available, or follows the cost of building new facilities, as in the infrastructure component modelled above. This applied only to Conflict and Vulnerable countries, where data on the number of damaged or destroyed facilities, as well as cost of repair, was publicly available. For the cost

---

[22] While revenue-raising is an important part of health financing, it has negligible incremental costs for this exercise, as its costs should be borne outside the health sector.

[23] The primary health care performance initiative. Primary Health Care Vital Signs Profiles: Detailed Methodology Note [Internet]. 2018. Available from: https://improvingphc.org/sites/default/files/VSP_Detailed_Methodology_Note.pdf

[24] Sands, P., Mundaca-Shah, C., & Dzau, V. J. (2016). The neglected dimension of global security—a framework for countering infectious-disease crises. New England Journal of Medicine, 374(13), 1281-1287.

[25] World Bank. 2012. People, Pathogens and Our Planet : The Economics of One Health. Washington, DC. © World Bank. https://openknowledge.worldbank.org/handle/10986/11892 License: CC BY 3.0 IGO.

of emergency relief, in any country that is still recovering from an emergency or conflict, an estimate of additional resource needs was estimated, comprised of a hazard pay packet given to health workers, estimated in proportion to the share of the population affected by conflict or emergencies. This applied only to Conflict and Vulnerable countries.

The costs of emergency preparedness and risk mitigation were more comprehensive. These included investments for disaster preparedness and emergency management, as well as those required for compliance with the International Health Regulations (IHR). These include laboratory capacity, the creation of Emergency Management teams within Ministries of health, with functional emergency operation centers for coordinated response, poison control centers, and national action plans for emergency preparedness, among others. The scale-up of laboratories is linked to the scale-up of hospitals, with laboratories being modelled as being built as part of every new district and provincial hospital, as well as prat of every refurbishment of district or provincial hospitals. National reference laboratories are built and scaled up in countries where there is no such laboratory, and built up over 5 years, with different starting times, depending on the country type.

No costs for the actual response to emergencies was included, as no forecasting along the expected annual emergencies, and their related cost, for each country was available at that time, and would have been necessary to accurately represent these costs. However, many components that are necessary to allow for an effective response, that every country should have in place in advance of emergencies, such as emergency kits, mobile lab kits, and mass casualty management supplies, are included.

3.7.2 Identifying PHC costs for Emergency Risk Management within the WHO 2017 SDG model

The costs for reconstruction and emergency relief are essential investments for a vulnerable or conflict affected country to get its health system back in order and delivering health service. They are therefore included in their entirety for PHC M1.

Among the estimated resources for emergency preparedness and risk management, there are none which are essential to the delivery of PHC services, except for laboratory services. Therefore, only a share of laboratory related costs are included in M1. The share applied is the same as the share used for infrastructure – i.e., 33% of district hospital level laboratories, and 3% of provincial level laboratories, but 0% costs of national reference laboratories).

Within M2, as the scope of services included under PHC is expanded, we include investments made within the other components. These include compliance with the International Health Regulations (IHR, 2004), national planning for health security, national and regional emergency operation centers, all-hazard training for health workers, and essential supplies and kits for being better prepared for responding to emergencies, such as body bags for mass-casualty management. The share of the costs of laboratories are also increased to include a larger share of each level. Here we apply the same share as for health facility infrastructure costs for inpatient care services.

3.8. Emergency Relief in distressed settings, and Facility Reconstruction in post-conflict settings

3.8.1.Context and previous resource estimates

Again, we draw on estimates developed within WHO's SDG price tag.

3.8.2 Identifying PHC costs within the WHO 2017 SDG model

Costs for Emergency Relief are included for 15 countries and essentially refer to hazard pay for health workers delivering services in distressed settings. Facility Reconstruction in post-conflict settings was estimated for six countries. We include these costs in their entirety under the proposed measures for all three measures of PHC.

Section 4: Methods for projecting health impact

For the majority of interventions, impact was estimated using projection models embedded within the OneHealth Tool. For other interventions we used Excel based models and/or drew on published literature.

Impact projections from the OneHealth Tool

The OHT is developed within Spectrum which is a suite of models that aim to provide policymakers with analytical tools to support priority setting and decision making processes. As such, OHT incorporates a variety of impact estimation models – including the Lives Saved (LiST) tool, the FamPlan model, and a number of models for Non-Communicable Diseases, – in order to project the costs and health impacts of scaling up specific interventions and activities in a given country. The key added value from projecting service coverage within the OHT is the linkage of separate disease impact projection models through a central demographic model, which ensures that deaths averted are not "double counted" but also allows us to benefit from the interaction of the interventions on different indicators in the tool (an example being a change in fertility rates from family planning affecting the number of children in need of a measles vaccination, and the impact of policies and regulation to reduce tobacco smoking and salt intake resulting in lower incidence, prevalence and mortality).

Modelling deaths averted

The methods for modelling deaths averted have been described elsewhere (Stenberg et al, 2017). Here we include only deaths averted from the range of interventions included under each measure. Thus, for example, water and sanitation interventions only have impact in measure M3.

The impact of family planning on deaths averted

Table S8 below illustrates the important impact that family planning has in terms of averting deaths that would otherwise have occurred in a business-as-usual scenario. Through the OneHealth Tool, we can extract the number of stillbirths, newborn and maternal deaths averted through increased coverage of health interventions. At the same time, we generate the total number of deaths averted by comparing the number of deaths that would have happened in a flatline scenario where coverage of all interventions remained constant over time, compared to the scale-up scenarios. This way, we can identify the reduction in modelled mortality arising from the modelled increase in family planning coverage, which ranges from 73% to 87% for the three PHC measures.

Table S8. Deaths averted from family planning vs health service scale-up, scenarios corresponding to three PHC measures, 67 low and middle-income countries

**Deaths averted, in millions (2020-2030)**	**PHC M1**	**PHC M2**	**PHC M3**
Newborn deaths averted			
From family planning and preventing unplanned births	7.9	7.9	7.9
From service scale-up	1.8	3.1	3.2
Stillbirths averted			
From family planning and preventing unplanned births	7.6	7.6	7.6
From service scale-up	0.3	2.2	2.2
Maternal deaths averted			
From family planning and preventing unplanned births	0.8	0.8	0.8
From service scale-up	0.4	0.6	0.6
Sum (newborn + maternal + stillbirths)			
From family planning and preventing unplanned births	16.3	16.3	16.3
From service scale-up	2.5	6.0	6.0

Modelling increases in life expectancy

Summary measures of health such as life expectancy, healthy life expectancy and healthy life years gained provide a general assessment of country progress towards strong primary care and universal health coverage. The OneHealth Tool (OHT) projections, including Spectrum impact modules (AIM, GOALS, LIST, DemProj, FamPlan, NCD), produce estimates on changes to population and deaths by age, taking into account coverage of interventions to prevent or treat various diseases. Estimates on life expectancy were calculated in Excel, drawing upon outputs from Spectrum/OHT. The Spectrum model tracks the population by single age as people are born, grow older, and die, and produces outputs on modelled deaths by age. We used these outputs to adjust/construct standard life tables[26] to estimate life expectancy at birth, and drawing upon GBD2010 disability weights by region,[27] to calculate the healthy life years gained due to scale up of interventions

Comparing the life expectancy under the scale-up scenario to the projected life expectancy with a constant coverage scenario, allows us to estimate the LE gained through the scale-up of the interventions, whilst implicitly taking into account the background projected increase in LE built-into the UN pop projections.

The 2030 projected life expectancy at birth within the scale-up scenarios includes the impact of scaling up care HIV/AIDS, maternal and child health, and a set of non-communicable diseases (cardiovascular disease, diabetes, asthma, COPD), epilepsy, and mental, neurological, and substance abuse disorders, as modelled through the OHT. Additional data available for Cancers, TB and NTDs were available from models with the same underlying methodology which we were able to incorporate into the calculations.

[26] Life tables: http://www.who.int/healthinfo/statistics/LT_method.pdf?ua=1&ua=1 WHO methods and data sources for life tables 1990-2015 (Global Health Estimates Technical Paper WHO/HIS/IER/GHE/2016.8)

[27] For Disability weights, see Salomon et al. (2012).

As with the SDG price-tag analysis, we additionally explicitly show the impact of avoiding still births on life expectancy increases. Intrapartum and Antepartum stillbirths are counted differently to avoided deaths following a live birth. A body of literature suggests that sentience begins at 28 weeks gestation, thus we would consider the fetus as a being from this point in time and would therefore include these data in health gain calculations.[28] Although sentience exists, there appears to be consensus that each stillbirth avoided should not be valued the same as neonatal death following live birth.[29] Thus, each intrapartum stillbirth avoided is weighted at 75% and each antepartum stillbirth avoided is weighted at 25% of a neonatal death. Antepartum: Each Antepartum stillbirth is valued at 0.25 of a death and life in the life expectancy calculations. In Healthy life expectancy, we assume the same health adjustment as per a neonatal life saved. For healthy life years gained, we apply 25% of the healthy life years gained for a neonatal death avoided.

We estimate the modelled increase in life expectancy between the 2015 baseline and the scale-up scenarios, which captures a broader increase in LE due to the underlying increase in general population health as captured within the UN pop projections (LE_B). In departure from the previous analysis, we were now able to run projections of life expectancy and healthy life years gained for all 67 countries included in the analysis. For this analysis we compared only LE_B, i.e. the increase in life expectancy at birth from 2015 to 2030 attributable to scale up of PHC interventions.

[28] Quereshi, Z U (2015) ; Phillips and Millum, (2015).
[29] Jamison DT, Shahid-Salles SA, Jamison J, et al.(2006)

Section 5. Constructing Investment guide posts

The guide posts were developed to signal incremental or total investment needs for PHC. Within our model, information is more readily available for measures related to Measure 1 than for the other two proposed measures.

5.1. Investment guide posts for expenditure

Current expenditure on PHC is based on Option 5 from the Van de Maele et al (2018) paper, and includes the following:

- General outpatient curative care, general dental curative care, home-based curative care, long-term outpatient and home-based car
- Preventive care
- Medical goods provided outside of healthcare services (typically purchased in pharmacies)
- A share of the health system governance services, based on the percentage of PHC over the current health expenditure (CHE).[30]

We would argue that a measure of PHC should also include ancillary and rehabilitation services. However the measures examined by Van de Maele et al do not consider an option which includes rehabilitation services and at the same time excludes specialized outpatient care. Thus, for our purpose we have applied option 5 as designed by Van de Maele et al and for which the Global Health Expenditure Database (GHED) reports expenditure on PHC.[31]

As of June 2019, data on PHC expenditure was available from the WHO GHED for 45 low and middle-income countries. Data was reported in USD2016 for year 2016. We deflated the estimates to USD2014 by using country specific price deflators drawn from the GHED. Thus we could estimate population weighted average expenditure on PHC per capita for three income groups, as shown below.

Guide posts	Measure	LIC	LMIC	UMIC
Current per capita spending on PHC	Recurrent expenditure only	24.9	33.9	304.3

We calculate the investment guide post for additional PHC expenditure per capita (investment guide post Expenditure Additional, or IGP-EA) for each country i, as per the three measures of PHC, with and without capital.

Next, to construct the investment guide post for total PHC expenditure per capita (investment guide post Expenditure Total, or IGP-ET) for country i , we would need to add the country-specific additional estimated per capita cost for year 2030 from our model to the average expenditure estimate for 2016 (EXP) for country group j.

$IGP\text{-}ET_i = EXPj + IGP\text{-}EA_i$

Here we do not report country specific estimates, but rather by country group. Thus, we construct the investment guide post for total PHC expenditure per capita (IGP-ET) for country group j as the regional aggregate population-weighted additional per capita cost for year 2030 as compared to 2015 (IGP-EA), plus the country-group average expenditure estimate for 2016 (EXP_j).

$IGP\text{-}ET_j = EXPj + IGP\text{-}EA_j$

---

[30] As information on the share of PHC/CHE is not available for all countries, an 80% share is applied for governance and financing related costs.

[31] WHO (2018) Indicators of the Global Health Expenditure Database 12 5 2018 https://apps.who.int/nha/database/DocumentationCentre/Index/en Accessed 31 July 2019.

Admittedly the incremental costs are additional to 2015 whereas the expenditure is estimated for 2016. However as described above, for our model we assume that little or no progress was made between 2015 and 2016 for the majority of investments modelled.

5.2. Investment guide posts for outpatient visits

We extracted the number of outpatient visits per intervention from our modelled projection files within the OneHealth Tool, by country and year. The numbers consider the population in need for each intervention, the specified coverage target, and the average number of outpatient visits required per intervention based on WHO recommended procedures and treatment protocols. The number of outpatient visits are calculated by delivery platform (community, outreach, first level clinic, and referral/hospital level). We summed the total number of visits per platform. Results are aggregated per country income group and per country health system classification.

Below is shown the total estimated number of outpatient visits in 2030 for the package of services modelled in the OneHealth Tool for the three measures of PHC. Estimates vary across country categories. This is because of varying disease burden across countries and different coverage targets set for 2030 within the model (see section 1).

The numbers derived can be compared with the reported number of outpatient contacts per person per year in European Union countries, which is 7.6 contacts per person per year (range 2.2.-12.1).[32] The limitations of the model used is that the model scope is partial and does not include a full set of what can be considered PHC services. On the other hand, the model does not always consider that the multiple interventions can be delivered during the same visit and may thus overestimate the number of contacts required

Table S9. Projected number of outpatient visits for three proposed measures of PHC service packages; OneHealth Tool simulations across 67 low and middle-income countries

	M1		M2		M3	
	Total OPVs per capita (2030)	Incremental OPVs per capita (2015-2030)	Total OPVs per capita (2030)	Incremental OPVs per capita (2015-2030)	Total OPVs per capita (2030)	Incremental OPVs per capita (2015-2030)
**Health System Typology**						
Conflict	3.7	2.7	3.8	2.8	3.6	2.6
Vulnerable	5.7	4.1	5.7	4.1	5.3	3.7
HS1	5.7	3.9	5.7	3.9	5.3	3.5
HS2	4.0	2.3	4.1	2.3	3.9	2.2
HS2	8.9	3.5	9.0	3.6	8.9	3.5
**By income group**						
LIC	5.7	4.0	5.8	4.1	5.4	3.7
LMIC	4.0	2.3	4.1	2.3	3.9	2.2
UMIC	8.7	3.5	8.8	3.6	8.7	3.5

Notes to table: OPV = outpatient visit

The number of additional visits is less for M3 than for M2, because M3 includes interventions that reduce exposure to risk factors, notably water and sanitation.

[32] Source: https://gateway.euro.who.int/en/indicators/hfa_543-6300-outpatient-contacts-per-person-per-year/ Accessed 31 July 2019.

Figure S1. **Projected additional outpatient visits needed to scale up PHC-M1 per capita, by service delivery platform; OneHealth Tool simulations across 67 low and middle-income countries**

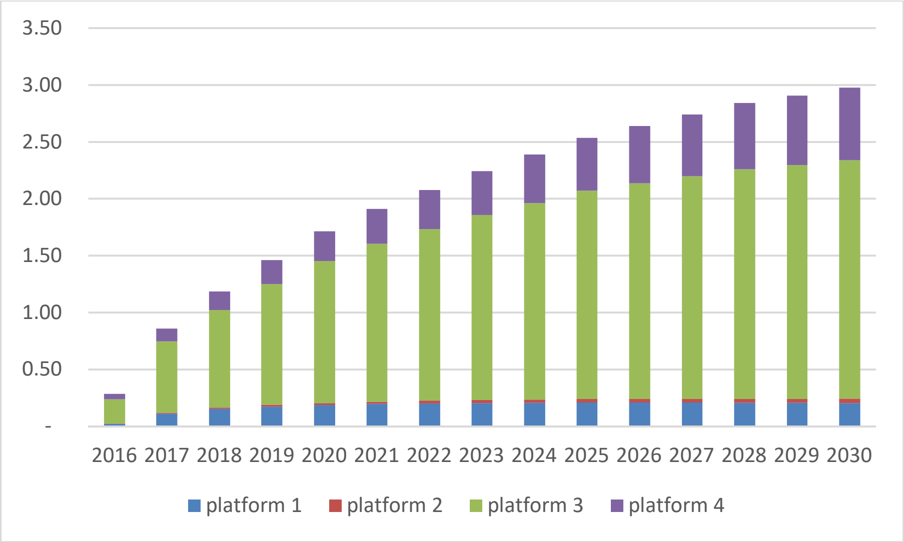

Platform 1: Policy and population wide interventions; Platform 2: Periodic outreach services; Platform 3: First level clinical services; Platform 4: First level and above

5.3. Investment guide posts for health workforce

Using the method described above in section 3.2, we estimated the number of additional health workers by country and year. The total number of additional health workers was summed by country group, and then divided by the total population in 2030 for countries within that group. Results are shown below. Low-income countries have a higher estimated need for other health workers because this category includes community health workers which are modelled to be required in greater density in rural settings.

Table S10. Health worker-population ratios derived from the OneHealth Tool simulations for three proposed measures of PHC services, 2030, by country group

	Medical Doctors	Nurses/Midwives	Other categories	Sum
**Measure 1**				
LIC	0.77	2.74	2.43	5.95
LMIC	1.03	3.04	1.97	6.04
UMIC	1.95	3.07	3.06	8.08
Conflict	0.94	2.92	2.13	5.99
Vulnerable	0.67	2.76	2.39	5.82
HS1	0.86	2.86	2.49	6.21
HS2	0.98	2.94	1.87	5.79
HS3	1.95	3.03	3.05	8.04
**Measure 2**				
LIC	0.99	2.93	2.78	6.70
LMIC	1.17	3.28	2.55	6.99
UMIC	1.99	3.30	3.16	8.44
Conflict	1.16	3.13	2.52	6.81
Vulnerable	0.95	3.00	2.77	6.71
HS1	1.04	3.03	2.82	6.89
HS2	1.12	3.18	2.45	6.75
HS3	1.98	3.25	3.13	8.36
**Measure 3**				
LIC	0.99	2.84	3.27	7.10
LMIC	1.17	3.24	3.32	7.73
UMIC	1.99	3.30	3.29	8.57
Conflict	1.16	3.09	2.93	7.17
Vulnerable	0.95	2.90	3.11	6.96
HS1	1.04	2.94	3.52	7.50
HS2	1.12	3.14	3.22	7.48
HS3	1.98	3.25	3.24	8.46

Note to table: Ratios are calculated as health workers per 1,000 population.

Section 6. Methods for estimating available financing

This section describes the methods used to produce projections of health expenditure under different scenarios to estimate the potential available resources and financing gap to advance PHC.

Outputs

The projections cover the period from 2017 to 2030 and include the overall envelope of current health expenditure (CHE)

Inputs

The key variables and sources used in the projections are shown in the below table

Acronym	Variable	Source
CHE	Current health expenditure	WHO Global Health Expenditure Database
GGE	General government expenditure	IMF, World Economic Outlook, April 2019
GDP	Gross domestic product	IMF, World Economic Outlook, April 2019
POP	Population	UN World Population Prospects 2017, Total Population Medium Fertility Variant

The health expenditure variables are based on the international system of health accounts (SHA2011), which defines the boundaries of health spending and separates current and capital health expenditure. Due to limited data availability and characteristic fluctuations in capital investments, our projections focus on the current health expenditure envelope (i.e. recurrent health spending).

The macro economic variables used in the health expenditure projections and financing gap analysis come from the IMF. Due to the absence of a complete and comparable set of projections to 2030 for all countries, we applied the non-parametric method of bootstrapping to obtain growth estimates of gross domestic product (GDP) and general government expenditure (GGE) beyond the IMF's projections to 2024 (further details provided below).

Scenarios

We developed country-specific projections of current health expenditure based on three possible scenarios for growth in spending:

1. Trend (business as usual following the historical trend of each country)
2. Progress (achieving a 1% point increase in CHE%GDP)
3. Ambitious (achieving a 2% point increase in CHE%GDP)

Units of Measurement

All amounts are measured in constant 2014 USD. Exchange rates are held constant for future periods based on 2016 annualised exchange rates. All aggregated results are calculated as unweighted simple averages unless otherwise indicated.

Methods

Simple and transparent estimation methods were adopted to enable a common approach for all countries using only universally available inputs such as population models and gross domestic product forecasts. We have used range estimates over point estimates because of the inherent uncertainty associated with long-term projections.

Current health expenditure (CHE)

CHE for each country for years 2017 to 2030 in each scenario is given by:

$$CHE_{c,t} = \left[\left(\frac{CHE}{GDP}\right)_{c,\ t-1} \times f(\delta_c) \right] \times GDP_{c,t}$$

where

- c is the country and t is year
- GDP is gross domestic product based on IMF-WHO projections
- CHE is current health expenditure
- $f(\delta_c)$ is a health-economy expansion function

This projection gives the overall envelop of health spending as a function of GDP and the share of health expenditure to the total economy. This relationship between health expenditure and economic development has been studied extensively and is often modelled as an elasticity including multiple predictive variables. In the absence of such data for all countries, a simple and transparent model was adopted.

The business as usual scenario applies a health-economy expansion function based on country specific historical trends given by linear regression of observed health expenditure data from 2000 to 2016. The progress and ambitious scenarios are based on normative increases in CHE as a share of GDP. Specifically, a 1% point increase over 2016 to 2030 under the progress scenario and a 2% point increase under the ambitious scenario (e.g. CHE as % of GDP increases from an initial 3% to 4% under progress scenario and 5% under ambitious scenario). This assumed expansion of CHE as a share of GDP under the progress and ambitious scenarios is generally consistent with theoretical literature and observed historical trends.

Although this expansion of CHE creates the potential for increased health care access, the quality, efficiency and equity of a country's health care system are also central. As described below under limitations, this analysis does not look at the breakdown of health spending including public and out of pocket expenditure. Consistent with the spirit of UHC and Alma-Ata, it is envisaged that the expansion of CHE would come primarily from public sources. This emphasis on government spending is also consistent with the Addis Ababa Action Agenda, which focuses on the need to raise domestic resources.

<u>GDP and GGE</u>

On the advice of counterparts from the World Bank (WB) and the International Monetary Fund (IMF), we prepared GDP and GGE growth projections for 2025 to 2030 with lower and upper ranges using the IMF's projections to 2024 and each country's historical data. The non-parametric method of bootstrapping to obtain expected average growth rates with a lower and upper bound for years 2025-2030 was adopted for its simplicity and its non-reliance on statistical assumptions about the normality of the data. The bootstrap was based on 1000 sample replications (draw and replacement) and greater weighting was placed on more recent years (2014-2024) assuming that current growth patterns would have more influence on future growth out to 2030. Specifically, the

$$W_i = i^{th} / \sum_{i=1}^{24} i \rightarrow 2000{:}\ W_1 = \frac{1}{325} = 0.0030 \quad 2002{:}\ W_2 = \frac{2}{325} = 0.0061$$

where

- w are weights of sampling probabilities (e.g. weight for 2000 observations = 0.3%, for 2001 =0.6% and so on)

A sensitivity analysis comparing the GDP growth rates obtained using the Bootstrap method with available GDP growth rates from other sources showed our projected range estimates to be consistent and robust.

<u>Limitations</u>

Projections of current health expenditure provide only the overall envelope of spending without specifying its breakdown into different components. How different revenue sources of health expenditure change in the future will be shaped by among other factors future health financing reforms, which will in turn impact on progress towards UHC in terms of access to quality services and financial protection.

One notable challenge not addressed is the uncertainty of future aid flows and allocations, which are highly variable and politically determined. Another important challenge is the incompleteness of data on capital investments, which can represent a large share of overall health spending. Characteristically, capital investments also have large fluctuations, which justifies analysing capital separately from current health expenditure. This calls for countries to better report capital investments and is a limitation when comparing projected current health spending with costs that include capital. Although the expenditure projections only include current health spending, this should not imply capital investments can be neglected. Indeed increases in health spending should go towards both current and capital expenditure since they are complementary.

Finally, it should be noted that the analysis relies on GDP and GGE growth projections that are inherently difficult to predict far into the future. To mitigate this, the study uses the best available forecasts from the IMF then builds upon these by constructing a range of average expected growth rates although this implicitly assumes the continuation of historical long-term growth.

Results

Here we present results that compare costs for Measure 1 with the projected available financing. PHC incremental costs for M1 increase from an initial 146 billion in 2020, 212 billion in 2025 and 224 billion in 2030. The average additional costs throughout the period 2020-2030 is 200 billion. In per capita terms, PHC incremental costs are estimated to increase from an average of 24USD in 2020 to an average of 32 USD per capita in 2030 (range 18USD-109USD).[33]

Figure S2. **Projected incremental costs for PHC M1, in billion US$ and average per capita US$, 67 low and middle income countries**

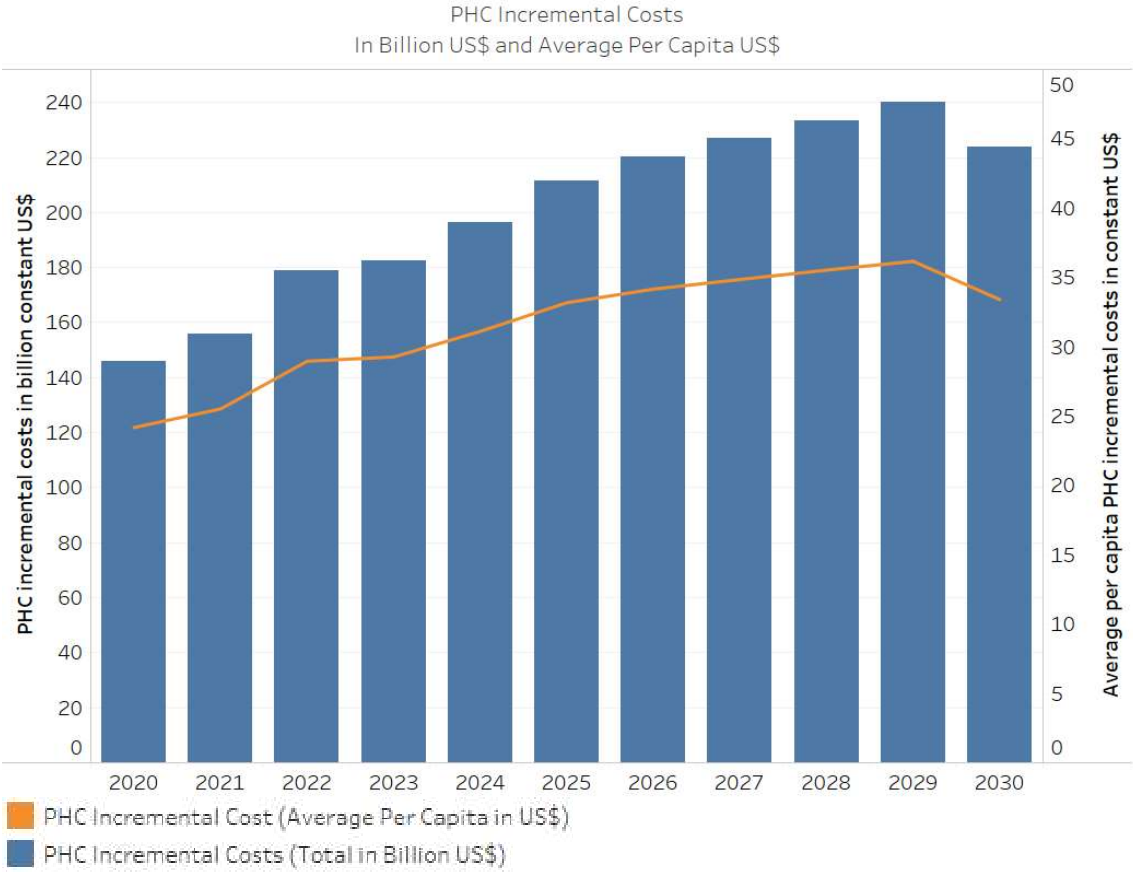

[33] Per capita averages are population weighted

In terms of affordability, these PHC M1 incremental costs in 2020 would represent on average 2.7% of forecast GDP (median 1.3% range 0.1% - 15.5%) and on average 12.5% of GGE (median 6.7% range 0.3% - 66.1%). In 2030, these PHC costs would represent on average 3.3% of GDP (median 1.7% range 0.1% - 20.2%) and 14.6% of GGE (median 6.7% range 3.3% - 71.3%).

Under the business as usual/trend scenario, CHE%GDP would increase from an average of 5.6% in 2016 to reach 6.1% (median 5.4% range 2.0%-17.8%) in 2020 and 6.6% (median 5.7% range 1.9%-21.4%) by 2030. Under the normative progress 1% and ambitious 2% scenarios, CHE%GDP would increase by these amounts respectively compared with the baseline 2016 values.

Figure S3. **Additional incremental costs for PHC M1, in billion US$ 2014, compared with projected additional health expenditure, total for 67 low and middle-income countries**

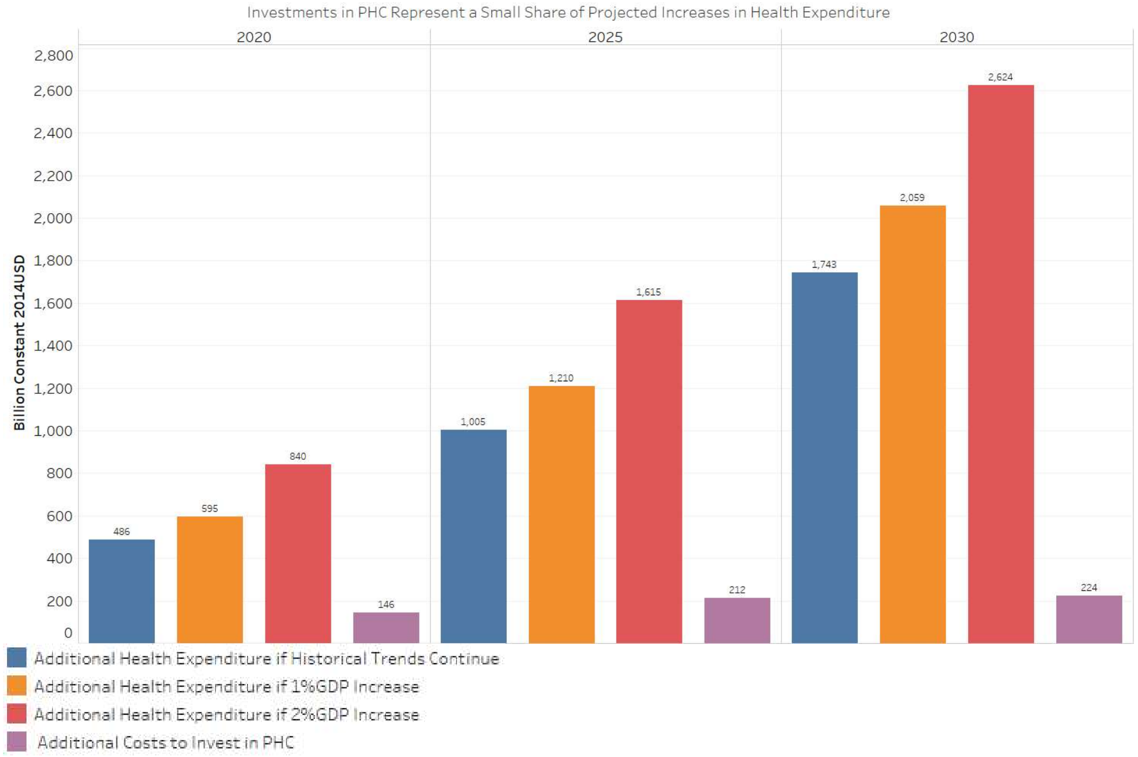

Figure S4. **Additional incremental costs for PHC M1, compared with projected GDP, per country**

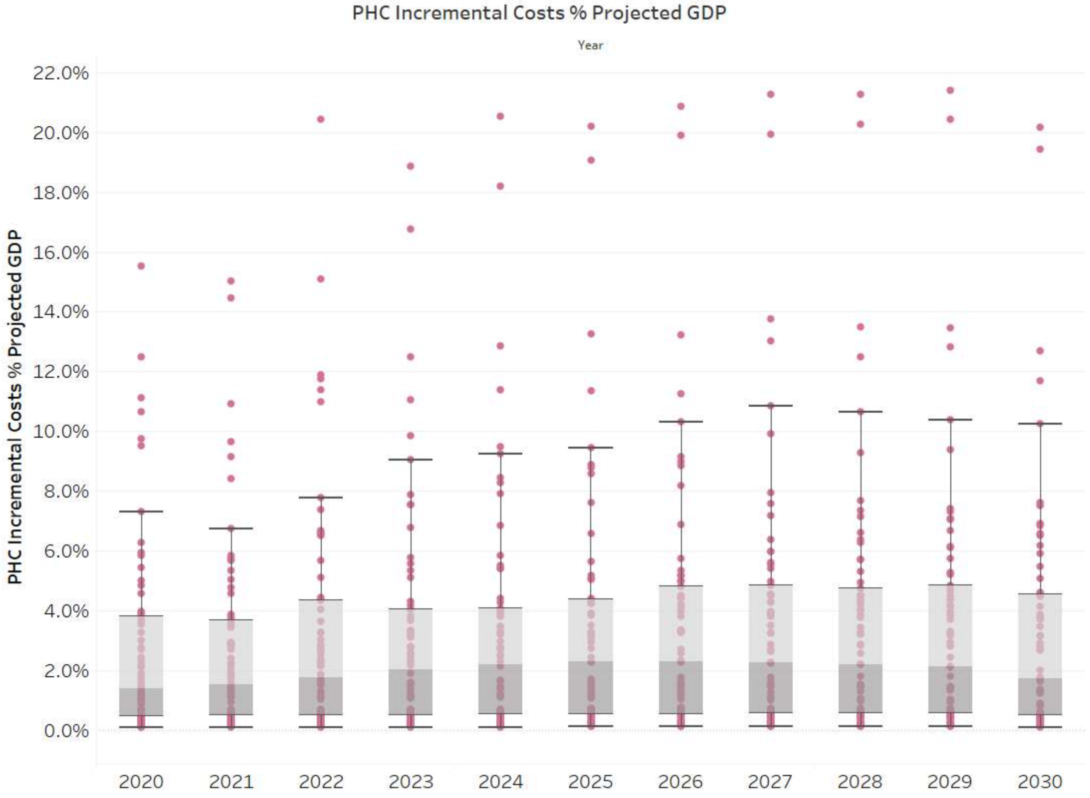

Box plot shows the interquartile range (25th–75th percentile) of values with the median marked by the join of the two coloured areas inside the bar. The lines and whiskers extend 1.5 times the interquartile range above and below the bar.

Comparing the financing scenarios, 29 countries (11 low income, 11 lower middle income, 7 upper middle income) are projected to achieve larger incremental available resources under the trend scenario compared to the 1% scenario and 16 countries (8 low income, 3 lower middle income, 5 upper middle income) are predicted to achieve larger incremental finances under the trend scenario compared to the 2% scenario. In all of these cases, business as usual/trend incremental available resources would exceed those of the 1% and 2% scenarios immediately from the beginning of the period of 2020 to 2030.

Under the business as usual/trend scenario, the gap in funding in 2030 is 35.7 billion USD of which 18.8 billion in low-income countries and 16.9 billion in low-middle income countries. In per capita terms in 2030, the average gap in funding is 32 USD (median 30USD, range 6USD - 130USD). Over the period 2020 to 2030, the number of countries with funding gap is projected to decrease from 40 to 25. Of these 25 countries, 18 are low income and 6 are lower-middle income. Under the trend scenario, incremental available resources would on average cover 50% of incremental PHC costs in the 25 countries still with a gap in 2030 (median 48%, range 10.5% -92.9%). The model predicts 38 countries with a gap of at least one of the years between 2020 and 2030.

Figure S5. **Number of countries with additional financing need even after increasing allocation towards PHC (i.e., gap between PHC M1 additional costs and projected additional finances)**

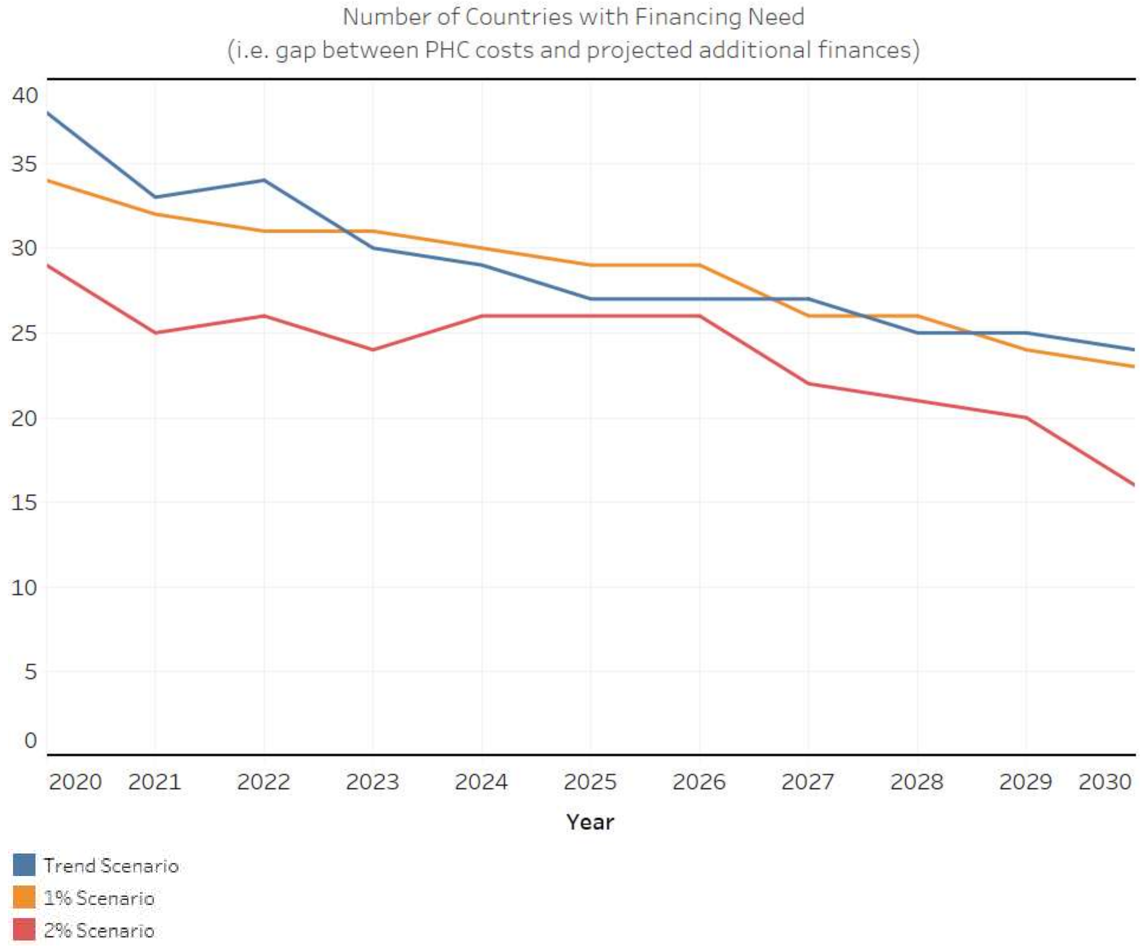

Under the ambitious 2% scenario, the gap in funding in 2030 is projected to be 11.3 billion of which 6.2 billion in low-income countries and 5.1 billion in low-middle income countries. In per capita terms in 2030, the average gap in funding is 28 USD (median 13 USD, range 6USD - 74USD). Over the period 2020 to 2030, the number of countries with funding gap in the model decreases from 28 to 15. Under the 2% scenario, incremental available resources covered on average 58% of incremental PHC costs in the 15 countries still with a gap in 2030 (median 71.3%, range 19.3% -82.8%). There are 29 countries that are predicted to have a funding gap of at least one of the years between 2020 and 2030.

Section 7. Consultation and review process

This work was guided by regular consultation and review processes. Significant work was undertaken to ensure the conceptual framework matched the measures that were being developed concurrently for PHC expenditure monitoring.

In September 2018, WHO organised an expert review and country feedback meeting to discuss the methodology and preliminary results of the analysis. Participants included international experts and academics, and representatives from 12 low and middle-income countries jointly accounting for more than 48% of the population covered in the model.[34]

The methods for estimating the resource needs for the SDG price tag had already been discussed at a previous meeting, and this meeting was set up to focus on discussing the proposed measures for PHC and their relevance at the global and country level. Participants considered the conceptual approaches for measuring investment needs for PHC and the components that should be considered within each PHC measure. Participants also reviewed and discussed preliminary results for costs related to the three measures, and discussed key messages.

Moreover, country participants reviewed country specific input assumptions to several components of this analysis, such as current population coverage levels of PHC interventions, and current numbers of health workers and health facilities. Country participants also reviewed prices used within the model for key inputs such as health worker salaries, and provided feedback on these. The main objective for the country-specific review was to update the inputs data for cost drivers, as well as to verify assumptions on current coverage which informs the health impact projections. Feedback was only incorporated into model revisions when reference documents were provided.

[34] Bangladesh, Brazil, China, Ethiopia, Indonesia, Iran, Mexico, Myanmar, Nigeria, Philippines, Sri Lanka, and Thailand.

Section 8. Additional results tables and figures

Table S11. Expenditure Trends by country

Income	Country	Health Spending USD Per Capita (2016)	GDP USD Per Capita (2016)	Health Spending % GDP (2016)	Population (Millions) (2016)	Health Spending Annualised % Change (2000-2016)	GDP Annualised % Change (2000-2016)	Government Spending Annualised % Change (2000-2016)
Low	Afghanistan	62	603	10.3%	35.4	8.0%	7.3%	18.0%
	Benin	37	948	3.9%	10.9	3.5%	4.1%	4.1%
	Burkina Faso	49	731	6.8%	18.6	10.6%	5.8%	6.7%
	Burundi	20	266	7.7%	10.5	4.6%	3.2%	2.2%
	Central African Republic	18	411	4.3%	4.5	-1.8%	-0.9%	-2.6%
	Chad	41	914	4.5%	14.6	5.9%	7.1%	5.6%
	Congo (DRC)	19	499	3.9%	78.8	11.6%	5.5%	16.8%
	Eritrea	36	1,227	3.0%	3.4	-1.4%	2.3%	-3.4%
	Ethiopia	25	623	4.0%	103.6	8.4%	9.0%	6.7%
	Gambia	18	623	2.9%	2.1	2.9%	3.1%	8.2%
	Guinea	45	859	5.3%	11.7	6.3%	4.0%	5.5%
	Guinea-Bissau	40	667	6.1%	1.8	1.8%	3.0%	2.0%
	Haiti	44	820	5.4%	10.8	-0.4%	1.2%	4.7%
	Liberia	73	8	9.6%	4.6	14.4%	2.7%	8.7%
	Madagascar	28	461	6.0%	24.9	3.4%	2.7%	1.9%
	Malawi	36	370	9.8%	17.2	11.7%	4.5%	9.2%
	Mali	34	897	3.8%	18.0	3.2%	5.1%	6.0%
	Mozambique	34	674	5.1%	27.8	9.2%	7.5%	10.5%
	Nepal	48	768	6.3%	27.3	7.6%	3.9%	7.3%
	Niger	27	433	6.2%	20.8	5.6%	5.6%	8.0%
	Rwanda	54	793	6.8%	11.7	11.0%	7.9%	9.0%
	Sierra Leone	96	579	16.5%	7.3	9.7%	7.3%	9.7%
	South Sudan	22	1,159	1.9%	10.8	-5.6%	-12.0%	7.3%
	Tanzania	43	1,069	4.0%	53.0	7.8%	6.4%	8.6%
	Togo	45	679	6.6%	7.5	8.3%	3.6%	8.0%
	Uganda	47	765	6.1%	39.6	5.2%	6.6%	6.5%
Low-Mid	Angola	129	4,968	2.6%	28.8	9.8%	6.7%	2.7%
	Bangladesh	32	1,335	2.4%	158.0	7.2%	6.1%	7.2%
	Cambodia	74	1,212	6.1%	15.8	7.2%	7.7%	10.4%
	Cameroon	76	1,615	4.7%	23.9	5.4%	4.3%	6.4%
	Comoros	68	889	7.6%	0.8	-0.8%	2.1%	6.3%
	Côte d'Ivoire	77	1,742	4.4%	23.8	1.6%	3.2%	5.1%
	Egypt	161	3,470	4.6%	94.4	3.9%	4.3%	5.8%
	Ghana	66	1,989	3.3%	28.5	7.0%	6.3%	9.3%
	India	65	1,802	3.6%	1,324.5	6.6%	7.3%	7.8%
	Indonesia	117	3,752	3.1%	261.6	8.7%	5.4%	6.0%

	Kenya	64	1,402	4.5%	49.1	4.5%	4.7%	7.2%
	Morocco	194	3,313	5.8%	35.1	6.9%	4.4%	5.8%
	Myanmar	72	1,334	5.4%	53.0	16.2%	9.2%	11.5%
	Nigeria	113	3,087	3.6%	186.0	9.1%	6.9%	0.7%
	Pakistan	37	1,330	2.8%	203.6	4.0%	4.3%	5.3%
	Philippines	137	3,112	4.4%	103.7	7.4%	5.3%	4.6%
	Sudan	120	2,022	5.9%	39.8	6.0%	2.5%	3.1%
	Tunisia	299	4,310	6.9%	11.3	5.4%	3.3%	4.1%
	Ukraine	186	2,760	6.7%	44.7	3.4%	1.9%	2.7%
	Uzbekistan	148	2,359	6.3%	31.4	8.5%	7.4%	5.0%
	Viet Nam	128	2,253	5.7%	93.6	7.5%	6.5%	8.0%
	Yemen		1,145		27.2		0.1%	-4.1%
	Zimbabwe	108	1,425	7.6%	14.0	2.5%	-0.3%	7.0%
Up-Mid	Algeria	376	5,643	6.7%	40.6	7.9%	3.6%	6.1%
	Azerbaijan	519	7,570	6.9%	9.7	13.3%	9.2%	13.9%
	Brazil	1,305	11,110	11.8%	206.2	6.2%	2.4%	3.4%
	China	423	8,500	5.0%	1,414.0	10.2%	9.4%	14.2%
	Colombia	488	8,315	5.9%	48.2	4.6%	4.1%	4.3%
	Dominican Republic	441	7,250	6.1%	10.4	6.4%	4.9%	6.1%
	Ecuador	510	6,099	8.4%	16.5	10.1%	3.9%	7.2%
	Iran	489	6,041	8.1%	79.6	6.9%	3.4%	4.6%
	Iraq	247	7,463	3.3%	36.6	9.9%	10.4%	-0.7%
	Kazakhstan	448	12,705	3.5%	17.8	5.6%	6.7%	6.3%
	Malaysia	459	12,067	3.8%	30.7	7.4%	4.8%	4.0%
	Mexico	619	11,331	5.5%	123.3	3.3%	2.0%	3.9%
	Peru	363	7,024	5.2%	30.9	6.0%	5.2%	5.1%
	Romania	547	10,976	5.0%	19.8	4.9%	3.9%	3.2%
	South Africa	515	6,338	8.1%	56.2	3.4%	2.9%	4.6%
	Sri Lanka	161	4,141	3.9%	21.0	4.8%	5.4%	4.5%
	Thailand	233	6,295	3.7%	69.0	5.2%	4.0%	4.6%
	Turkey	553	12,810	4.3%	79.8	4.5%	4.9%	4.1%

Source: WHO Global Health Expenditure Database, July 2019

Table S12. Additional estimated costs under three Measures of PHC (US$ 2014)

Estimate	Measure	Year	M1	M2	M3
a. Total additional cost (US$ billion)	Recurrent and capital	Sum 2020-2030	2188	2780	3631
b. **Annual** additional cost (US$ billion)	Recurrent and capital	Average 2020- 2030	200	253	328
c. **Annual** additional cost (US$ billion)	Recurrent only	Average 2020- 2030	179	238	300

Table S13. Percentage (%) share of SDG price tag costs included under the three Measures of PHC *

**Component**	**M1**	**M2**	**M3**
Infrastructure	47%	72%	72%
Health workforce	53%	71%	76%
HIS	20%	57%	472%
Supply chain	86%	105%	108%
Health financing policy	80%	100%	100%
Governance	80%	100%	100%
Emergency Risk Management (incl IHR)	9%	50%	50%
Commodities and supplies	88%	89%	88%
Emergency relief	100%	100%	100%
Reconstruction costs in conflict and fragile settings	100%	100%	100%
Additional health programme costs	99%	100%	100%

**Estimated as the share calculated for the full period (2016-2030) in the original 2017 model.*

Table S14. Country shares of additional cost for PHC as a percentage (%) share of total projected costs under each Measure (years 2020-2030)

	M1	M2	M3
Afghanistan	0.5%	0.3%	0.5%
Algeria	0.6%	0.6%	0.6%
Angola	1.4%	1.1%	1.3%
Azerbaijan	0.1%	0.5%	0.1%
Bangladesh	2.2%	1.4%	2.5%
Benin	0.3%	1.0%	0.3%
Brazil	1.6%	1.5%	1.6%
Burkina Faso	0.6%	0.4%	0.5%
Burundi	0.3%	0.4%	0.3%
Cambodia	0.4%	0.4%	0.3%
Cameroon	0.9%	0.7%	0.8%
Central African Republic	0.1%	0.4%	0.1%
Chad	0.4%	0.3%	0.4%
China	23.5%	18.4%	22.9%
Colombia	0.9%	4.3%	0.7%
Comoros	0.0%	0.2%	0.0%
Democratic Republic of the Congo	2.4%	1.3%	2.7%
Côte d'Ivoire	0.9%	1.8%	0.8%
Dominican Republic	0.2%	0.4%	0.2%
Ecuador	0.4%	0.3%	0.3%
Egypt	1.9%	1.2%	2.0%
Eritrea	0.2%	1.1%	0.2%
Ethiopia	3.2%	1.5%	3.0%
Gambia	0.0%	1.7%	0.0%
Ghana	0.7%	0.4%	0.8%
Guinea	0.3%	0.5%	0.3%
Guinea-Bissau	0.0%	0.2%	0.0%
Haiti	0.2%	0.1%	0.2%
India	11.4%	8.2%	12.6%
Indonesia	3.6%	6.5%	4.4%
Iran	1.4%	2.7%	1.2%
Iraq	2.3%	2.1%	1.3%
Kazakhstan	0.3%	0.6%	0.2%
Kenya	1.2%	0.7%	1.3%
Liberia	0.1%	0.6%	0.1%
Madagascar	0.6%	0.4%	0.7%
Malawi	0.4%	0.5%	0.5%
Malaysia	0.4%	0.6%	0.4%
Mali	0.4%	0.2%	0.4%
Mexico	1.5%	1.4%	2.0%

Morocco	1.2%	1.3%	1.1%
Mozambique	1.3%	1.0%	1.3%
Myanmar	0.7%	1.0%	0.6%
Nepal	0.5%	0.5%	0.5%
Niger	1.5%	1.3%	1.1%
Nigeria	4.9%	4.2%	6.1%
Pakistan	5.7%	4.5%	4.9%
Peru	1.0%	3.2%	0.9%
Philippines	1.2%	1.2%	1.5%
Romania	0.3%	0.8%	0.2%
Rwanda	0.2%	0.3%	0.3%
Sierra Leone	0.4%	0.4%	0.3%
South Africa	0.9%	0.9%	1.5%
South Sudan	0.3%	0.6%	0.3%
Sri Lanka	0.3%	0.3%	0.3%
Sudan	1.8%	1.5%	1.5%
United Republic of Tanzania	1.8%	1.3%	1.9%
Thailand	0.3%	1.3%	0.5%
Togo	0.4%	0.4%	0.3%
Tunisia	0.4%	0.3%	0.2%
Turkey	1.3%	0.7%	1.2%
Uganda	1.3%	1.3%	1.2%
Ukraine	0.4%	1.1%	0.4%
Uzbekistan	0.4%	0.2%	0.6%
Viet Nam	1.9%	1.5%	1.8%
Yemen	1.0%	1.4%	0.8%
Zimbabwe	0.5%	0.5%	0.5%

Table S15. Components of additional projected PHC cost, in billion US$2014 and as a percentage (%) share of projected costs under each Measure

Cost component	M1 - costs in billion	M1 - costs as %	M2 - costs in billion	M2 - costs as %	M3 - costs in billion	M3 - costs as %
Infrastructure	53.8	27%	81.3	32%	81.3	25%
Health workforce	61.9	31%	84.6	33%	90.1	27%
Health Information Systems	0.1	0%	0.3	0%	2.4	1%
Supply chain	10.4	5%	11.7	5%	13.3	4%
Health financing policy	1.1	1%	1.4	1%	1.4	0%
Governance	1.4	1%	1.8	1%	1.8	1%
Emergency Risk Management	0.1	0%	0.6	0%	0.7	0%
Emergency Relief and Humanitarian Relief	0.8	0%	0.7	0%	0.7	0%
Commodities	43.5	22%	44.1	17%	43.7	13%
Programme costs	25.7	13%	26.2	10%	26.2	8%
Costs outside health sector	N/A	N/A	N/A	N/A	66.7	20%
SUM	200	100%	253	100%	328	100%

Because of rounding, numbers might not add up

Figure S6. Projected average additional costs for PHC per capita 2020-2030, by country category, PHC Measure 1 *

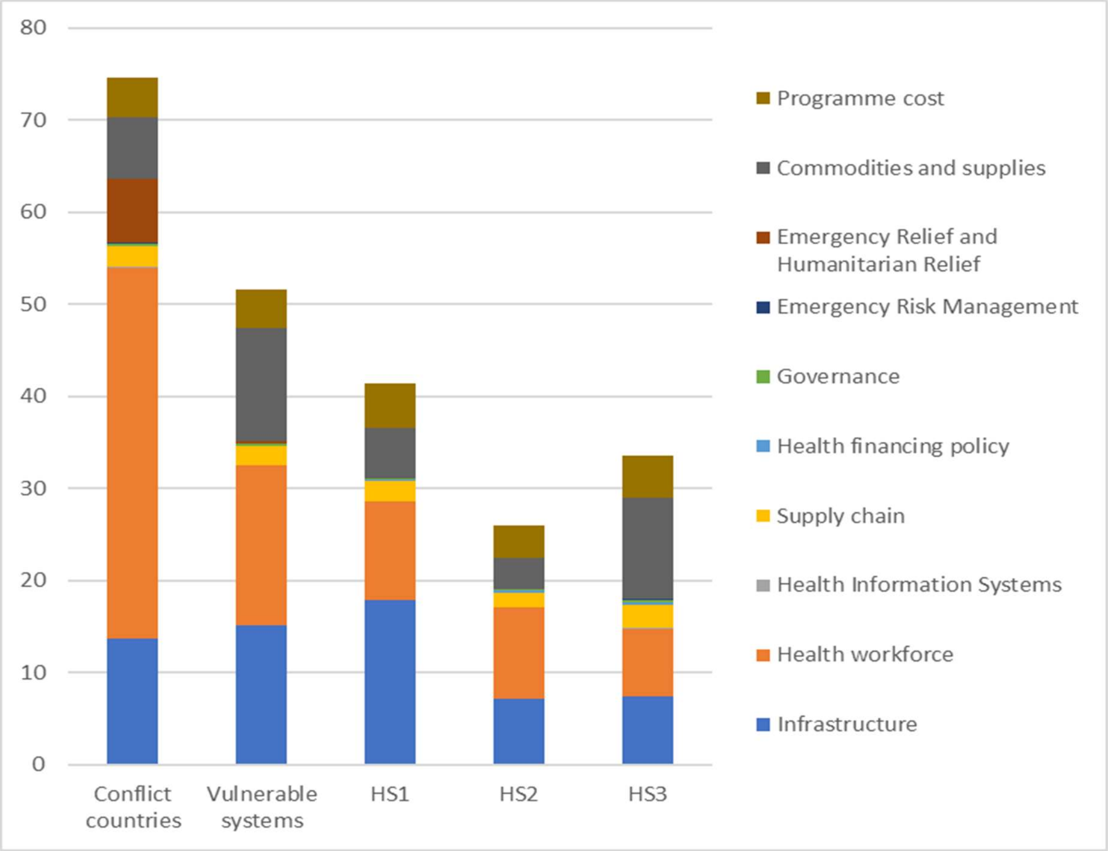

*Population weighted average per capita costs, average 2020-2030. For a description of country categories, see section 1.2.

References for supplementary material

OECD, Eurostat, WHO (2011), A System of Health Accounts, OECD Publishing. DOI: http://dx.doi.org/10.1787 McCord J, Tien M, Sarley D. Guide to public health supply chain costing: a basic methodology. Arlington, VA: USAID | DELIVER PROJECT, Task Order 4; 2013.

Jamison DT, Shahid-Salles SA, Jamison J, et al. Incorporating Deaths Near the Time of Birth into Estimates of the Global Burden of Disease. In: Lopez AD, Mathers CD, Ezzati M, et al., editors. Global Burden of Disease and Risk Factors. Washington (DC): The International Bank for Reconstruction and Development / The World Bank; 2006. Chapter 6. Available from: https://www.ncbi.nlm.nih.gov/books/NBK11805/ Co-published by Oxford University Press, New York.

MacKenzie, A., Murphy, G. T., & Audas, R. (2019). A dynamic, multi-professional, needs-based simulation model to inform human resources for health planning. Human resources for health, 17(1), 42.

Phillips, J., & Millum, J. (2015). Valuing Stillbirths. Bioethics, 29(6), 413–423. http://doi.org/10.1111/bioe.12120

Portnoy A, Ozawa S, Grewal S, et al., 2015. Costs of vaccine programs across 94 low- and middle-incomecountries. *Vaccine*. 2015 May 7;33 Suppl 1:A99-108. doi: 10.1016/j.vaccine.2014.12.037.

Qureshi, Z. U., Millum, J., Blencowe, et al. (2015). Stillbirth should be given greater priority on the global health agenda. The BMJ, 351, h4620. http://doi.org/10.1136/bmj.h4620

Salomon JA, Vos T, Hogan DR, et al. Common values in assessing health outcomes from disease and injury: disability weights measurement study for the Global burden of Disease Study 2010. 2012 The Lancet 380(9859): 2129-2143.

Siddiqi S , Masud TI , Nishtar S , et al . Framework for assessing governance of the health System in developing countries: gateway to good governance. Health Policy 2009;90:13–25. doi:10.1016/j.healthpol.2008.08.005

Stop TB partnership (2016), Global Plan to End TB: the paradigm shift 2016-2020. http://www.stoptb.org/global/plan/plan2/

Stenberg K, Hanssen O, Edejer TT-T, et al. Financing transformative health systems towards achievement of the health Sustainable Development Goals: a model for projected resource needs in 67 low-income and middle-income countries. Lancet Glob Health. 2017; 5: e875-e887

Stover J, Bollinger L, Izazola JA, et al. (2016) What Is Required to End the AIDS Epidemic as a Public Health Threat by 2030? The Cost and Impact of the Fast-Track Approach. *PLoS ONE* 11(5): e0154893. doi:10.1371/journal.pone.0154893

Tomblin Murphy G, Birch S, MacKenzie A, Bradish S, Elliott Rose A. (2016). A synthesis of recent analyses of human resources for health requirements and labour market dynamics in high-income OECD countries. Hum Resour Health. 4:59.

Vande Maele N, Xu K, Soucat A, et al. Measuring primary healthcare expenditure in low-income and lower middle-income countries. BMJ Glob Health 2019;4:e001497. doi:10.1136/ bmjgh-2019-001497

Westbrook JI, Amanda Ampt, Leanne Kearney and Marilyn I Rob (2008), All in a day's work: an observational study to quantify how and with whom doctors on hospital wards spend their time. Med J Aust 2008; 188 (9): 506-509. || doi: 10.5694/j.1326-5377.2008.tb01762.x

Westbrook JI, Duffield C, Li L, and Creswick NJ (2011) .How much time do nurses have for patients? A longitudinal study quantifying hospital nurses' patterns of task time distribution and interactions with health professionals BMC Health Serv Res. 2011 Nov 24;11:319. doi: 10.1186/1472-6963-11-319.

WHO (2009), High-level Taskforce on Innovative International Financing for Health Systems. Constraints to scaling up the health Millennium Development Goals: costing and financial gap analysis, background document for the Taskforce on Innovative International Financing for Health Systems. http://www.who.int/choice/publications/d_ ScalingUp_MDGs_WHO_report.pdf (accessed April 1, 2016).

WHO (2010). The World Health Report. Health systems financing: the path to universal coverage.

WHO (2015), Global Technical Strategy for Malaria 2016–2030.

WHO (2015), Tracking universal health coverage: first global monitoring report.

WHO (2015), Investing to overcome the global impact of neglected tropical diseases.

WHO Global Health Observatory. http://www.who.int/gho/en/ Accessed 24 May 2016.

United Nations, Department of Economic and Social Affairs, Population Division, World Population Prospects: The 2019 Revision, New York, 2019
